# Secondary Metabolism and Development Is Mediated by LlmF Control of VeA Subcellular Localization in *Aspergillus nidulans*

**DOI:** 10.1371/journal.pgen.1003193

**Published:** 2013-01-17

**Authors:** Jonathan M. Palmer, Jeffrey M. Theisen, Rocio M. Duran, W. Scott Grayburn, Ana M. Calvo, Nancy P. Keller

**Affiliations:** 1 Department of Medical Microbiology and Immunology, University of Wisconsin–Madison, Madison, Wisconsin, United States of America; 2 Department of Biological Sciences, Northern Illinois University, DeKalb, Illinois, United States of America; 3 Bacteriology Department, University of Wisconsin–Madison, Madison, Wisconsin, United States of America; Duke University Medical Center, United States of America

## Abstract

Secondary metabolism and development are linked in *Aspergillus* through the conserved regulatory velvet complex composed of VeA, VelB, and LaeA. The founding member of the velvet complex, VeA, shuttles between the cytoplasm and nucleus in response to alterations in light. Here we describe a new interaction partner of VeA identified through a reverse genetics screen looking for LaeA-like methyltransferases in *Aspergillus nidulans*. One of the putative LaeA-like methyltransferases identified, LlmF, is a negative regulator of sterigmatocystin production and sexual development. LlmF interacts directly with VeA and the repressive function of LlmF is mediated by influencing the localization of VeA, as over-expression of *llmF* decreases the nuclear to cytoplasmic ratio of VeA while deletion of *llmF* results in an increased nuclear accumulation of VeA. We show that the methyltransferase domain of LlmF is required for function; however, LlmF does not directly methylate VeA *in vitro*. This study identifies a new interaction partner for VeA and highlights the importance of cellular compartmentalization of VeA for regulation of development and secondary metabolism.

## Author Summary

In recent years there has been increased interest in bioactive small molecules produced by filamentous fungi. Members of the genus *Aspergillus* are prolific producers of natural products such as penicillin, the cholesterol lowering drug lovastatin, in addition to several toxins, the most famous being aflatoxin. The genetic regulation of fungal natural products is coupled with developmental differentiation through a conserved protein complex termed the velvet complex. The founding member of the complex, *velvet* (VeA), is a light-regulated protein that shuttles between the cytoplasm and nucleus in response to illumination. Once in the nucleus, VeA interacts with the putative methyltransferase LaeA to positively regulate production of secondary metabolites and with VelB to induce sexual development. We have identified a new interaction partner of VeA that has sequence homology to LaeA. The putative LaeA-like methyltransferase LlmF controls the subcellular localization of VeA in response to light, thereby regulating the downstream outputs of secondary metabolism and development. While the mechanism of the velvet complex remains an enigma, our data suggest that manipulation of protein subcellular localization is an approach that can be used to control production of secondary metabolites.

## Introduction

Small bioactive secondary metabolites are molecules that have great importance to humankind and can be broadly characterized by their impact on human health. Members of the filamentous fungal genus *Aspergillus* are prolific producers of secondary metabolites, both useful (penicillin, lovastatin) and detrimental (aflatoxin, sterigmatocystin, gliotoxin), therefore they are excellent organisms to study the genetic regulation of secondary metabolism.

Genes responsible for the production of secondary metabolites are often clustered on chromosomes, vaguely reminiscent of bacterial operons, and these gene clusters are co-regulated [1]. Relatively recently a global regulator of secondary metabolism, LaeA, was described in the Aspergilli [2]. LaeA has since been shown to be part of the velvet complex, composed of LaeA, VeA, VelB, that couples secondary metabolism with developmental processes including asexual and sexual development [3]. Moreover, LaeA has been found to direct formation of a second velvet-like complex composed of VelB and VosA [4]. Both complexes function to orchestrate secondary metabolism with developmental differentiation in a light dependent fashion [3]–[5].

The core components of the velvet complex are found in all filamentous fungi studied to date and function to regulate important pathways, including pathogenicity in both plants and humans. For example, null mutants of *A. fumigatus laeA* produce fewer secondary metabolites and are hypovirulent in the mouse model of invasive aspergillosis [6], [7] while both VeA and LaeA homologs in the plant pathogens *A. flavus, Fusarium fujikuroi, F. verticillioides*, and *Cochliobolus heterostrophus* are involved in regulation of virulence and toxin production [8]–[12]. Recent work in *F. graminearum* has identified that both the *veA* and *velB* homologs are virulence factors [13]–[16], however these data indicate the heterotrimeric LaeA-VeA-VelB complex might be slightly different in this organism, as FgVeA did not interact with FgLaeA or FgVelB in a yeast-two-hybrid assay [13], [15]. Taken together, the velvet complex control of secondary metabolism and development appears to be conserved in filamentous fungi [5], however its cellular operational mechanism remains an enigma.

Current knowledge of the velvet complex functionality in *Aspergillus* centers on the importance of VeA as a bridging factor between VelB and the putative methyltransferase LaeA. Together, this heterotrimeric complex regulates developmental differentiation in response to light. LaeA is a constitutively nuclear protein that harbors a methyltransferase domain required for function [2], [17] and VelB is a velvet–like protein that forms a heterodimer with VeA [3]. In *A. nidulans*, VeA-VelB is required for sexual development, while VeA-LaeA is required for production of the mycotoxin sterigmatocystin [3].

An important aspect of VeA is its subcellular localization in response to illumination. Stinnett et al. [18] first reported that VeA is found in the cytoplasm under light conditions and is mainly nuclear under dark conditions, while the truncated VeA1 is blind to light and fails to accumulate in the nucleus in the dark. It has also been demonstrated that transcription of *veA* is relatively constant over all developmental stages [19] and this correlates with protein levels [4]. Therefore, the subcellular location of VeA has the potential to control protein interaction partners as well as to direct developmental and chemical responses to environmental cues.

While VelB-VeA-LaeA has been shown to be a stable complex that interacts in the nucleus, VeA interacts with several other proteins, including the red light sensing protein FphA, and thus indirectly with the blue light sensing proteins LreA and LreB [20], [21]. The VeA-family of proteins (VeA, VelB, VelC, VosA) all contain a characteristic velvet domain [5], while VosA also has a characterized C terminal transcriptional activation domain [5], [22]. Bayram and Braus [5] have speculated that the velvet domain may function as a protein-protein interaction domain. Calvo [23] and others (i.e. [5]) hypothesize that VeA could function as a scaffold protein directing transcriptional response to changes in environmental cues, such as illumination. Working under this hypothesis, the velvet complex may contain additional accessory proteins that could influence development and secondary metabolism.

We used a reverse genetics approach to identify several putative methyltransferases in *A. nidulans* that have sequence homology to LaeA, and then set out to determine if any of these putative methyltransferases were linked to the velvet complex. Here we describe the LaeA-like methyltransferase, LlmF, that is a negative regulator of sexual development and secondary metabolism in *A. nidulans*. We report that LlmF interacts with VeA and functions to mediate subcellular localization of VeA. This study identifies a new interaction partner of VeA while illustrating the importance of cellular compartmentalization as a form of regulating secondary metabolism and development.

## Results

### *In silico* identification of putative LaeA-like methyltransferases

**Figure 1:**
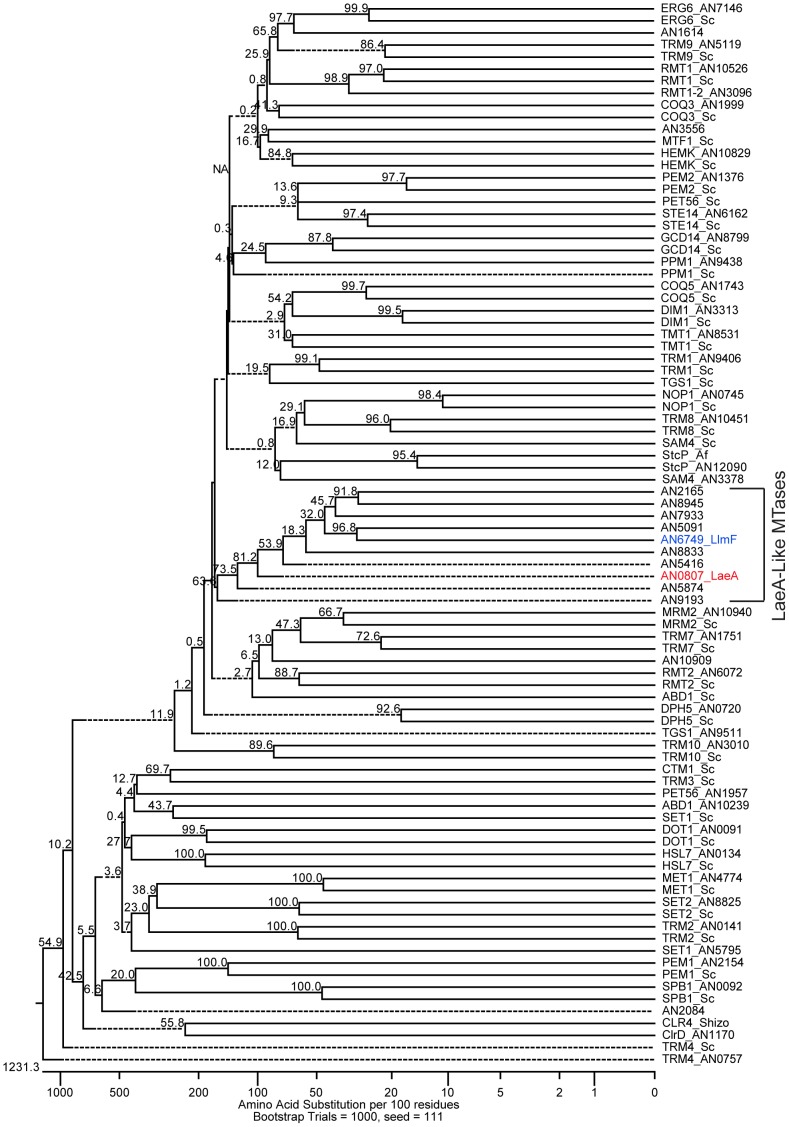
Reverse genetics identified LaeA-like methyltransferases in *A. nidulans*. Putative methyltransferases were identified from the genome annotation of *A. nidulans* at the Broad Institute, based on the presence of a predictive *S*-adenosyl methionine (SAM) binding domain. A ClustalW multiple sequence alignment was preformed on 88 amino acid sequences that corresponded to 50 identified from the *A. nidulans* genome, 37 previously characterized from *Saccharomyces cerevisiae* and *Schizosaccharomyces pombe*, and one from *A. flavus* (StcP). Bootstrapping analysis was done using the neighbor joining method with 1000 trials and a seed of 111 (MegAlign, DNASTAR), the numbers at each node indicate bootstrap values, and dashed lines represent collapsed nodes. This analysis identified nine uncharacterized loci that are homologous to LaeA. These nine loci have been named LaeA-like methyltransferases. AN5091 (LlmE) has previously been published and therefore is not included in this manuscript [24].

Since LaeA has been shown to regulate many but not all secondary metabolite clusters and VeA has several interaction partners, including LaeA, VelB, and FphA [23], we hypothesized that other LaeA-like methyltransferases could play a role in velvet complex developmental regulation. The primary amino acid sequence of LaeA (AN0807) indicates that it contains an *S*-adenosyl methionine (SAM) binding domain [2] that is required for function [17]. To determine if there are other putative LaeA-like methyltransferases in *A. nidulans*, predicted amino acid sequences were obtained for all SAM binding domain proteins from the *Aspergillus nidulans* genome database at the Broad Institute and aligned with known methyltransferases from *S. cerevisiae*, *Sc. pombe*, and *A. flavus*. A bootstrapped phylogenic tree was inferred from this analysis (Figure 1). This analysis identified nine putative ORFs that have sequence homology to LaeA and no homologies to previously characterized yeast methyltransferases (LlmA – AN2165, LlmB – AN8945, LlmC – AN7933, LlmD – AN5416, LlmE – AN5091, LlmF – AN6749, LlmG – AN5874, LlmI – AN8833, and LlmJ – AN9193). One of these putative ORFs, *llmE* (AN5091), has previously been characterized and resides in a location that is transcriptionally repressed by telomere position effect [24].

### Preliminary characterization of LaeA-like methyltransferases

**Figure 2:**
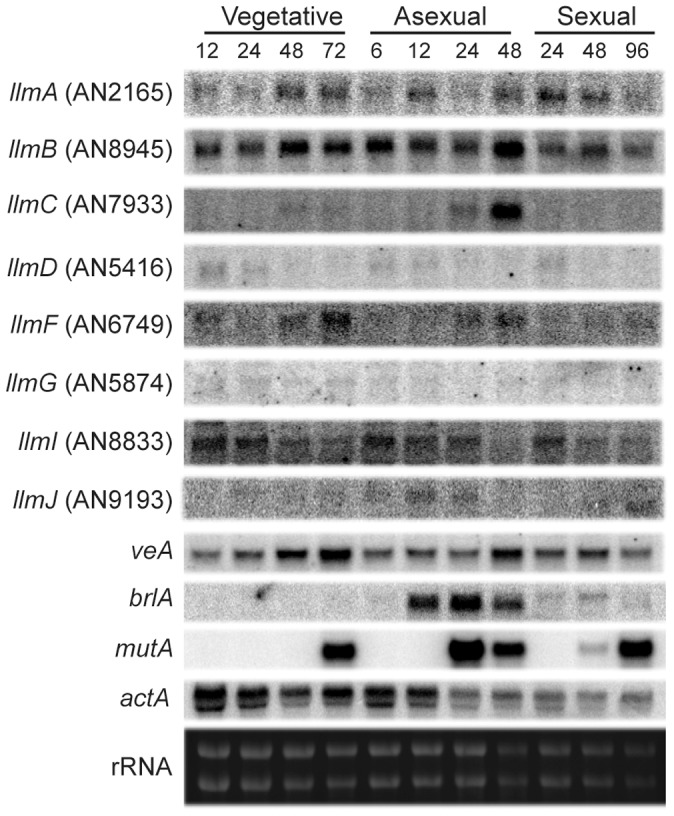
LaeA-like methyltransferases expression over different development stages. Expression profiling of all of the LaeA-like methyltransferases via northern blot indicates that some are expressed at very low levels (*llmD, llmG*, and *llmJ*), some are expressed constantly through development (*llmA, llmB*, and *llmI*), and *llmC* is expressed only during late asexual development. Controls for developmental time points were as follows: *brlA* was used as a control for asexual induction, *mutA* for sexual development, and actin (*actA*) as a loading control. Expression of *llmF* increases during both late vegetative and asexual development, however it does not increase during sexual development.

**Figure 3:**
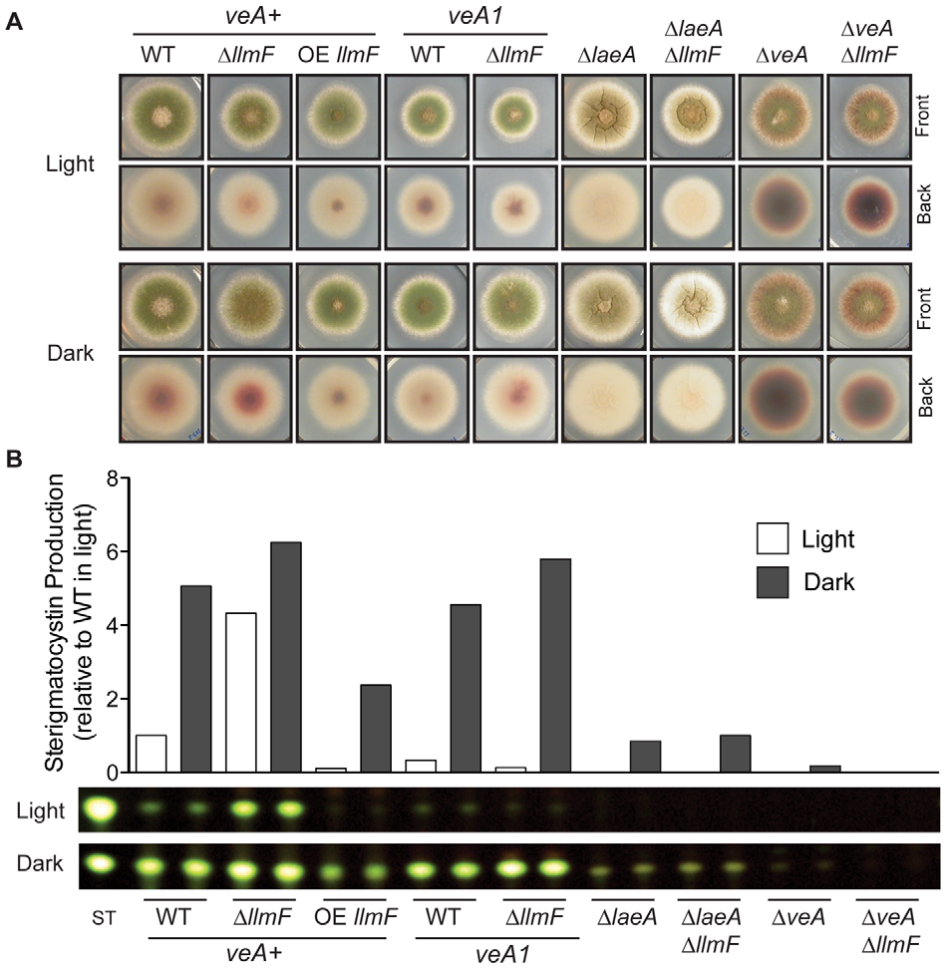
LlmF is a negative regulator of sterigmatocystin production. (A) Colony growth phenotypes are shown from strains grown on glucose minimal media (GMM) for 3 days in either light or dark conditions. (B) Overlay inoculated cultures were grown in either the light or the dark and 3 cores were taken from each plate after 4 days of growth at 37°C. The cores were homogenized in water and extracted with an equal volume of ethyl acetate. Thin layer chromatography using toluene: ethyl acetate: acetic acid (8∶1∶1) as a solvent of organic extracts followed by spraying with 15% aluminum chloride allows for the analysis of sterigmatocystin. Quantification of sterigmatocystin was achieved using ImageJ software.

In order to ascertain functions of the LaeA-like methyltransferase (Llm) proteins, transcriptional profiling from a wild type *A. nidulans* strain (RDIT9.32) was conducted at several distinct developmental growth time points (Figure 2). Northern analysis of the *llm* genes show that some are transcribed at nearly all developmental stages (*llmA, llmB*, and *llmI*), while others are transcribed at very low levels (*llmD, llmF, llmG*, and *llmJ*), and *llmC* is mainly transcribed during late asexual development (Figure 2). To assess the roles of each Llm on secondary metabolism, null mutants were created for all of the putative Llm's and confirmed by Southern blot (Figure S1 and data not shown). Preliminary characterization of secondary metabolite production indicated that Δ*llmF* produced increased levels of sterigmatocystin (Figure 3), whereas the other eight Llm's had minor impacts on sterigmatocystin (Figure S2, [24]). Therefore we chose to focus our efforts on characterization of LlmF (AN6749). While *llmF* appears to be expressed at low levels throughout development, there is an increase in *llmF* transcription during later stages of both vegetative and asexual growth conditions, while there is no increase of *llmF* during sexual development (Figure 2).

### LlmF is a negative regulator of sterigmatocystin

To gain further insight into *llmF* regulation of secondary metabolism, *llmF* was over-expressed with the constitutive *gpdA* promoter (data not shown). We assessed sterigmatocystin production in the *llmF* mutants, and oppositely to Δ*laeA* mutants, deletion of *llmF* results in increased sterigmatocystin levels. Conversely, over-expression (OE) of *llmF* reduced sterigmatocystin production (Figure 3). Interestingly, we did not detect an increase in sterigmatocystin production in a Δ*llmF* mutant harboring the *veA1* allele (Figure 3B). To test the requirement of LaeA and VeA in LlmF regulation of sterigmatocystin; double mutants were created and these analyses suggest that both LaeA and VeA are required for negative regulation of sterigmatocystin by LlmF (Figure 3).

### LlmF interacts directly with VeA

**Figure 4:**
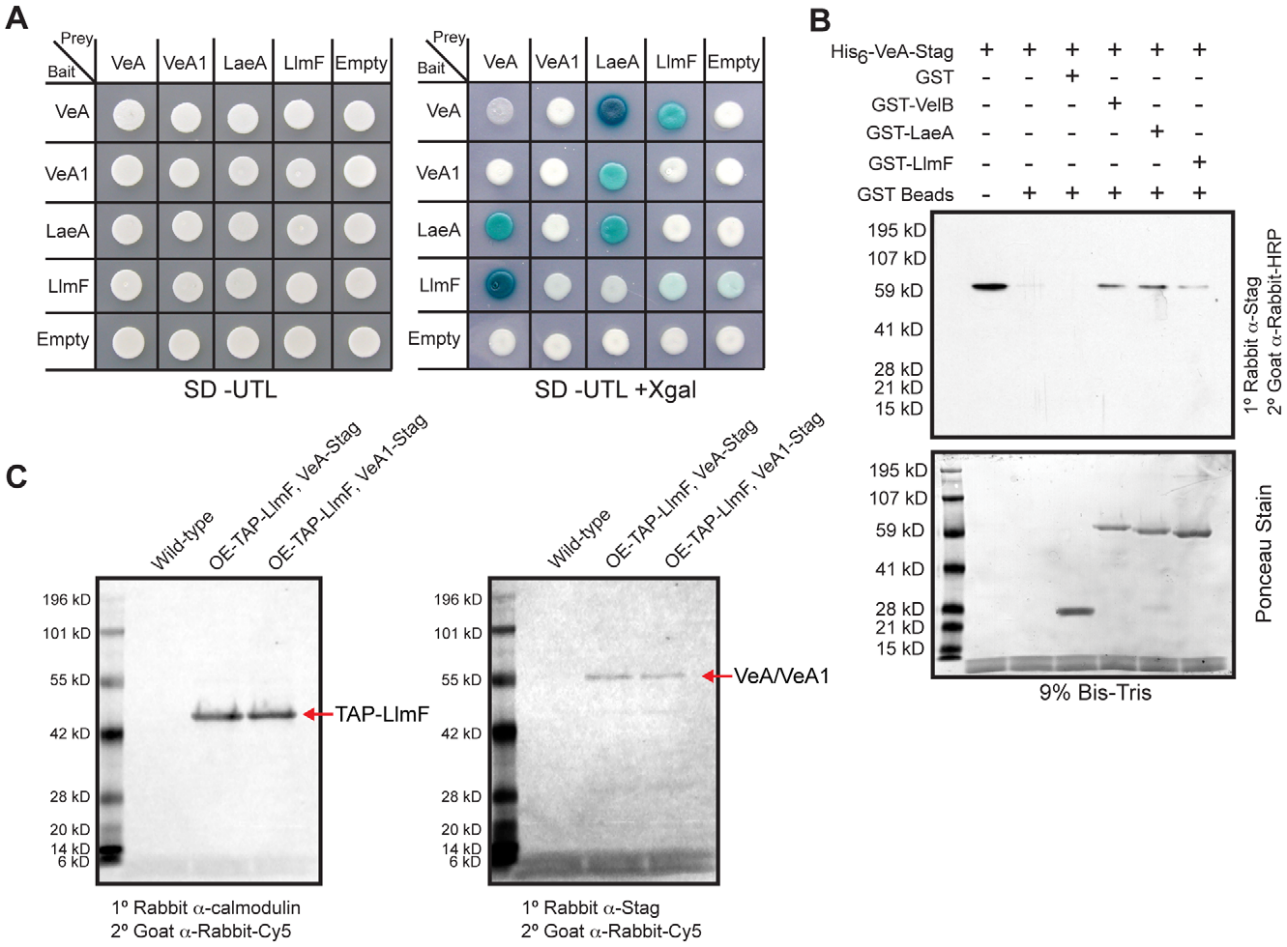
LlmF interacts with VeA in the yeast-two-hybrid, *in vitro* GST pull-down, and *in vivo* co-purification. (A) A directed yeast-two-hybrid approach measured protein-protein interactions and indicated LlmF interacts with VeA, but not the truncated VeA1. Yeast cells harboring the indicated bait and prey plasmids were grown in liquid shaking culture to a density of approximately 2×10^7^ cells/ml and 10 µl was spotted on synthetic dropout media (SD) containing the appropriate supplements (uracil (U), tryptophan (T), leucine (L), and/or X-gal). A positive interaction results in the activation of the *lacZ* reporter, which turns the media blue in the presence of X-gal. (B) Recombinant GST, GST-LlmF, GST-LaeA, and GST-VelB were incubated with recombinant His_6_-VeA-S-tag and subsequently purified by glutathione sepharose 6B to look for co-purification of VeA with any of the GST labeled proteins. An immunoblot using anti-S-tag antibody detected the presence of the His_6_-VeA-S-tag protein and Ponceau stain of the membrane served as an indication of the amount of GST fusion proteins in each lane. GST tagged LlmF, VelB, and LaeA were capable of pulling down His_6_-VeA-S-tag, while GST alone did not. (C) Crude protein extracts were prepared from one-liter liquid shaking culture of each strain and subjected to the TAP protein purification protocol. The resulting eluate was electrophoresed on a 10% Bis-Tris SDS-PAGE gel and transferred to a nitrocellulose membrane where an anti-calmodulin antibody confirmed TAP-LlmF and an anti-S-tag antibody was used to detect VeA-S-tag and VeA1-S-tag. Strains used are: WT =  RJMP103.5, OE-TAP-*llmF veA*-S-tag = RJMP249.1, and OE-TAP-*llmF veA1*-S-tag = RJMP250.2.

Since LlmF mediated regulation of sterigmatocystin was dependent on the velvet complex, we hypothesized that LlmF could interact directly with LaeA or VeA. A cDNA was cloned for LlmF (Figure S3) and a directed yeast-two-hybrid approach was taken to ascertain protein-protein interactions. These results indicated that LlmF interacts with VeA but not the truncated VeA1 or LaeA (Figure 4A). However, the yeast-two-hybrid assay is historically prone to false-positives and in addition we have noticed inconsistencies with VeA1 in this assay, for example Bait-LaeA does not interact with Prey-VeA1 (Figure 4A). Therefore, we also assessed the LlmF-VeA interaction with an *in vitro* GST pull-down assay with recombinantly expressed GST-LlmF and His_6_-VeA-S-tag (Figure 4B). As an assay control, GST-VelB and GST-LaeA were also used to pull-down His_6_-VeA-S-tag (Figure 4B). Finally, we performed an *in vivo* pull-down experiment using tandem affinity purification (TAP) tagged LlmF combined with S-tagged VeA and VeA1. After purification of TAP-LlmF, both VeA-S-tag and VeA1-S-tag were detected via immunoblotting (Figure 4C). Unlike the yeast-two-hybrid results, the *in vivo* pull-down data suggest that LlmF interacts with both VeA and the truncated VeA1 under these conditions.

### LaeA and LlmF can bind *S*-adenosyl methionine

**Figure 5:**
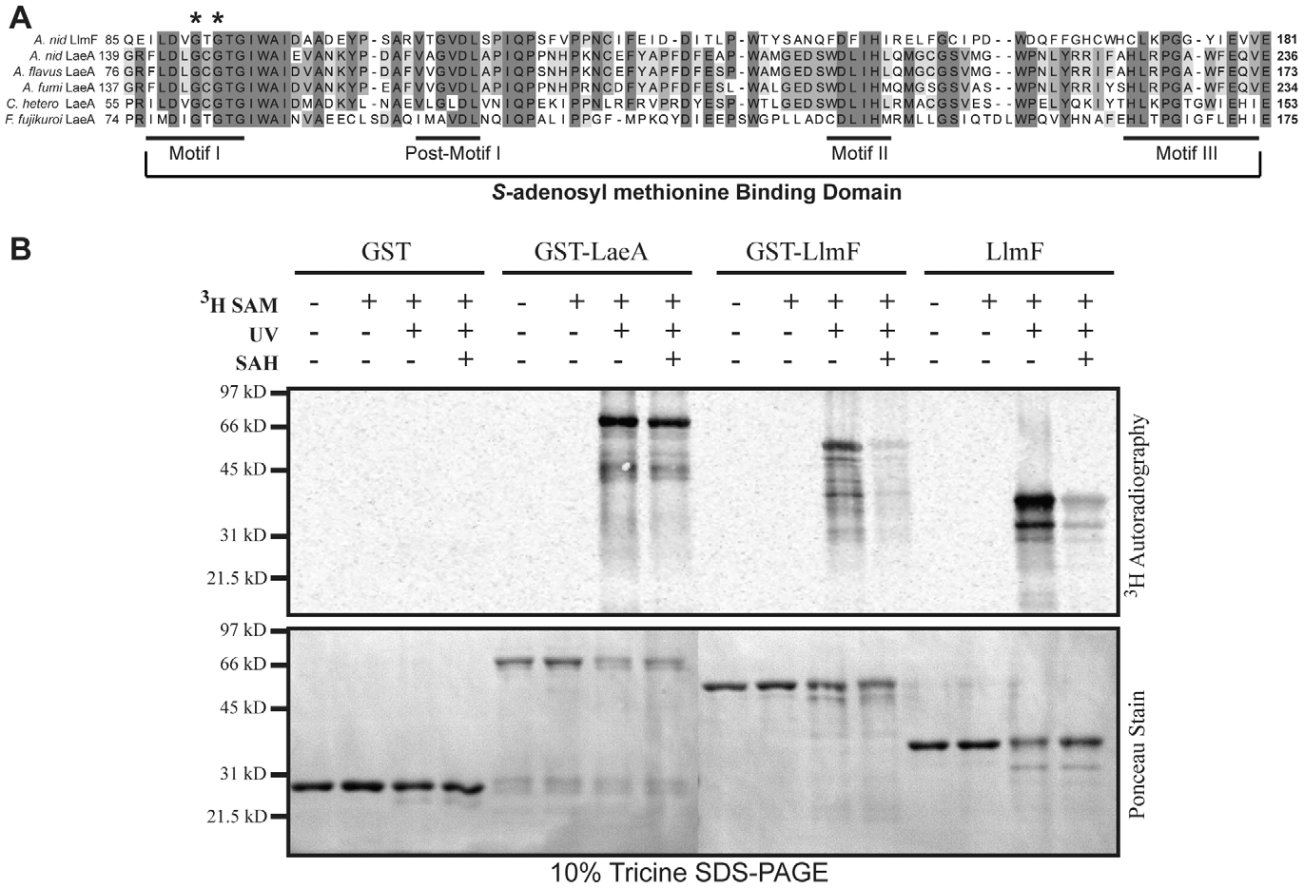
Recombinant LlmF binds the methyl donor molecule *S*-adenosyl methionine. (A) Multiple sequence alignment of LlmF with previously characterized LaeA proteins from *A. nidulans*, *A. flavus*, *A. fumigatus*, *Cochliobolus heterostrophus*, and *Fusarium fujikuroi* identifies that LlmF harbors a conserved *S*-adenosyl methionine (SAM) binding domain [26], [61]. The SAM binding domain can be further broken down into four motifs that correspond to β-strands in the binding pocket: motif I, post-motif I, motif II, and motif III. LlmF and LaeA proteins contain these conserved motifs. Asterisks indicate the conserved glycine residues in motif I that were previously mutated in LaeA to render the protein inactive [17]. (B) An ultraviolet (UV) crosslinking assay was used to test the SAM binding site prediction and show that both LaeA and LlmF are capable of binding SAM. ^3^H-SAM was incubated with recombinantly purified GST, GST-LaeA, GST-LlmF and LlmF for 30 minutes at room temperature. To crosslink ^3^H-SAM into the binding site of the enzyme, some samples were incubated on ice while being exposed to UV light for 30 minutes and then all samples were subsequently electrophoresed on a 10% Tricine SDS-PAGE gel, transferred to nitrocellulose membrane, and then exposed to a tritium phosphor screen. Active site crosslinking of SAM was confirmed by incubation with the active site competitive inhibitor, *S*-adenosyl-homocysteine (SAH) at a concentration of 1 µM.

Previous studies on LaeA had indicated that it was a likely methyltransferase based on site directed mutagenesis of the *S*-adenosyl methionine (SAM) binding domain, which resulted in a non-functional protein [17]. A multiple sequence alignment [25] of *A. nidulans* LlmF, *A. nidulans* LaeA, *A. flavus* LaeA, *A. fumigatus* LaeA, *Cochliobolus heterostrophus* LaeA, and *Fusarium fujikuroi* LaeA confirm that all of these proteins contain a SAM binding domain which can be further classified into conserved motifs as previously described [26], [27] (Figure 5A). We tested the functionality of the SAM binding domain by using an ultraviolet (UV) light cross-linking experiment of ^3^H-SAM (Figure 5B). As a control, GST and GST-LaeA were included in the experiment and these results indicate that both LaeA and LlmF have the ability to bind SAM (Figure 5B). Competitive inhibition of the binding site was achieved by co-incubation of ^3^H-SAM with *S*-adenosyl homocysteine (SAH) and under these conditions a reduction in ^3^H-SAM binding was observed in both GST-LlmF and LlmF. GST-LlmF but not GST-LaeA showed a reduction in ^3^H-SAM binding under these conditions, which could suggest that GST-LaeA binds SAM more tightly than LlmF.

### The SAM binding domain of LlmF is required for negative regulation of sexual development

In order to assess the role of the SAM binding site of LlmF, we constructed a SAM binding site mutant *in vivo* by site directed mutagenesis of motif I (Figure 5A). Mutation of two conserved glycine residues to alanine residues in motif I rendered LaeA non-functional [17], therefore we mutated the same residues in LlmF (G91A and G93A). To analyze the effect of this mutation *in vivo*, *llmF^G91A,G93A^* (*llmF^SAM^*) was driven by the *gpdA* promoter in a Δ*llmF* background. As abnormalities in sexual development - ranging from cleistothecial morphological aberrancies to alterations in sexual to asexual spore ratios - are clearly and consistently observed in *veA*, *velB* and *laeA* mutants [4], [28], we investigated the impacts of *llmF* on sexual development.

**Figure 6:**
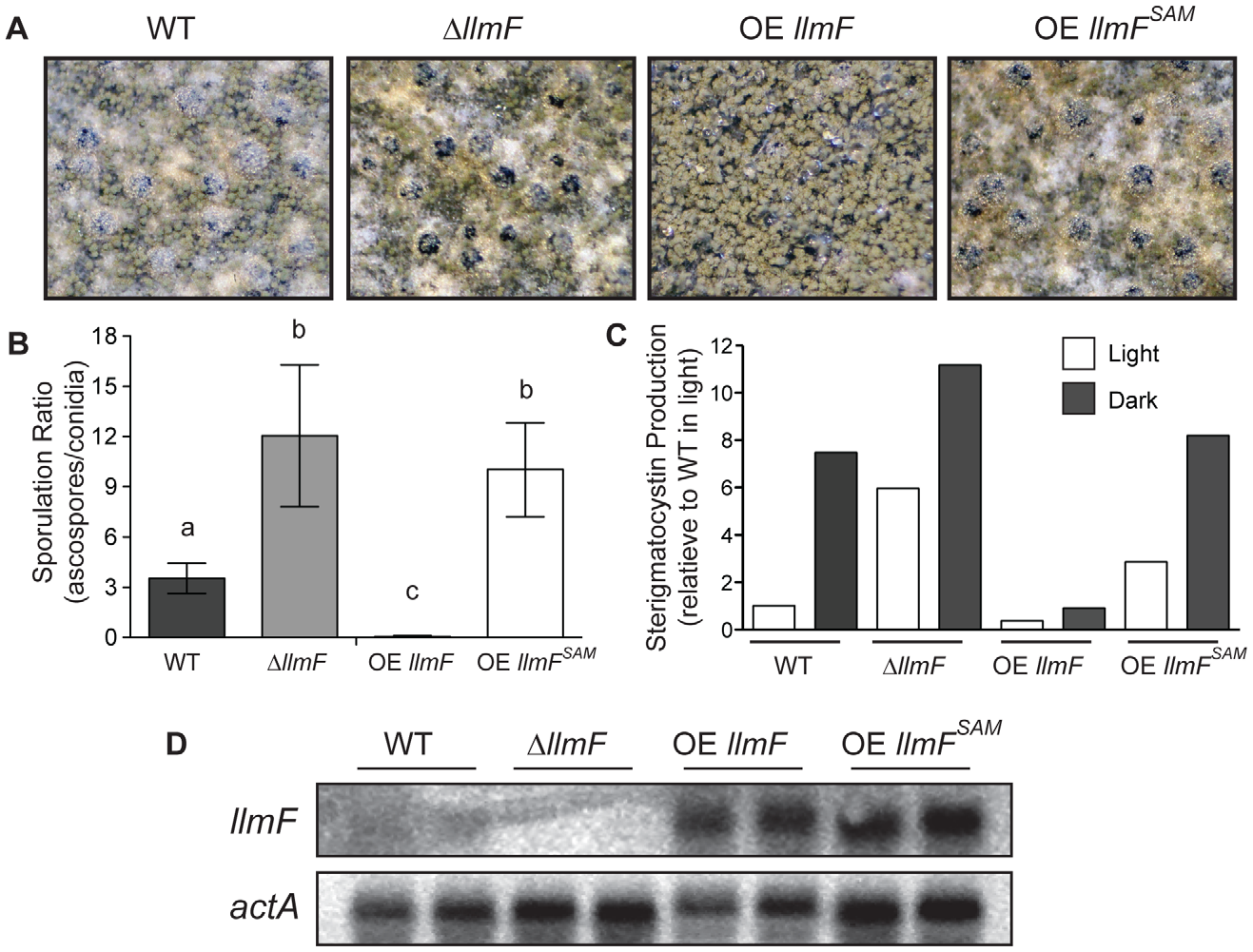
The methyltransferase domain of LlmF is required for negative regulation of sexual development and sterigmatocystin. (A) Cultures grown for five days under sexual developmental induction were imaged under a dissecting microscope. Wild type and Δ*llmF* strains produce abundant cleistothecia and few conidia, while OE *llmF* strains produce abundant conidia and few cleistothecia under these conditions. Additionally, the OE *llmF^SAM^* mutant displays a Δ*llmF* phenotype indicating the requirement of the SAM binding domain. (B) Quantification of spores produced under sexual developmental conditions (materials and methods) supports the macroscopic images, as the Δ*llmF* strain produces an increased ratio of ascospores to conidia (sexual to asexual) and the OE *llmF* strain produces a decrease in this ratio. These data demonstrate that the OE *llmF^SAM^* mutant has a sporulation ratio similar to that of the Δ*llmF* strain. Lowercase letters refer to statistical significance that measured with a student T-test of significance using Prism (Graphpad). (C) Analysis of sterigmatocystin was done in light and dark conditions as described in the materials and methods. Relative quantification of sterigmatocystin was achieved using ImageJ software and the bar graph indicates the average of two replicates. Notably, OE *llmF^SAM^* does not repress the production of sterigmatocystin that is displayed in the OE *llmF* strain. (D) Strains were confirmed by northern analysis of *llmF* and *actA* transcripts from RNA extracted from sexual developmental induction.

Macroscopic observations during sexual developmental induction of Δ*llmF* and OE *llmF* mutants showed that Δ*llmF* favored sexual development and OE *llmF* asexual development. Additionally, the OE *llmF^SAM^* mutant displayed a phenotype more similar to the Δ*llmF* mutant (Figure 6A). To more accurately measure *llmF* effect on sexual development, ascospores and conidia were quantified from sexually induced culture conditions and represented as a ratio of ascospores to conidia (Figure 6B). The Δ*llmF* strain produced an increased ratio of ascospores to conidia and consistent with the negative regulation of sexual development the OE *llmF* strain produces decreased ratio of ascospores to conidia. Mirroring the macroscopic images, the OE *llmF^SAM^* mutant produced a similar ratio to the Δ*llmF* strain (Figure 6B). Moreover, we measured production of sterigmatocystin from these four strains. This further confirms that LlmF^SAM^ is not functional as the OE *llmF^SAM^* mutant produced levels of sterigmatocystin intermediate to wild type and Δ*llmF*, in contrast to the reduced levels of the OE *llmF* strain (Figure 6C). Finally, a northern blot from total RNA isolated from the same conditions confirmed the strains were correct and the OE *llmF^SAM^* construct was properly expressed (Figure 6D). Taken together these data indicate that LlmF is a negative regulator of sexual development and secondary metabolism and the SAM binding site of LlmF is required for function.

### LlmF has limited effect on velvet complex transcription

**Figure 7:**
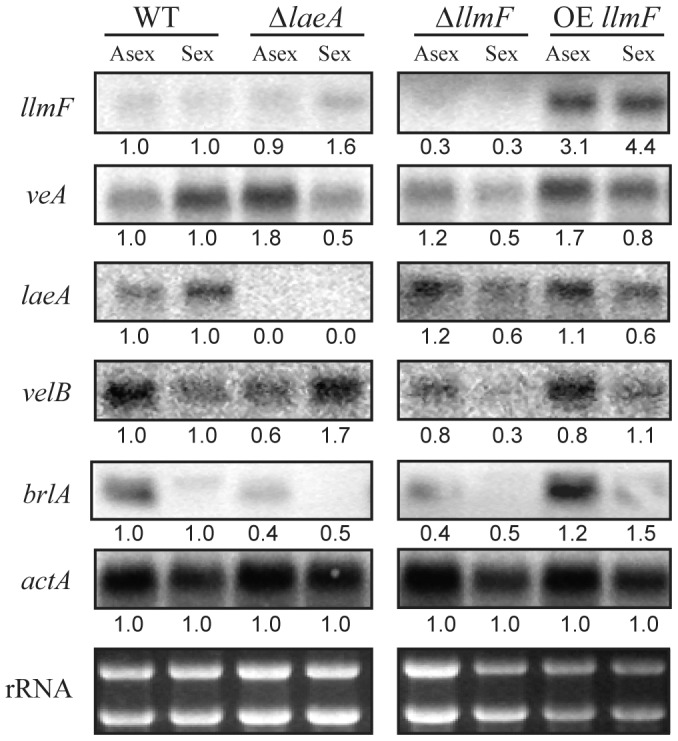
Expression of the velvet complex members is not increased in ΔllmF strains. Mycelia was grown in liquid shaking culture for 20 hours in minimal media and then subsequently transferred to solid minimal media plates. Asexual development was induced by incubation in constant light for 24 hours while sexual development was induced by incubation in constant darkness for 48 hours. Total RNA was extracted from these conditions and expression was analyzed with a northern blot. Actin (*actA*) was included as a loading control. Numbers represent relative expression levels that have been normalized to actin from each condition and then normalized to wild type expression for each growth condition (asexual or sexual). Quantification was done using ImageJ software. Strains are as follows: WT = RJMP144.6, Δ*laeA* = RJMP153.7, Δ*llmF* = RJMP144.9, and OE *llmF* = RJMP143.5.

Since the velvet complex has been implicated in transcriptional control of secondary metabolism and development, we hypothesized that the observed phenotypes could be explained by differential transcription of velvet complex members in the Δ*llmF* and OE *llmF* backgrounds. Specifically, we hypothesized that LlmF could negatively regulate transcription of *veA*, *laeA*, or *velB* which could explain the increased secondary metabolism and sexual development in the Δ*llmF* background. To test this hypothesis, a northern blot was done on RNA isolated from asexual and sexual developmental induced cultures. These data refute this hypothesis, as LlmF has minimal effect on transcription of *veA, laeA*, or *velB* (Figure 7). This is in contrast to the impact of loss or over-expression of velvet complex members on each other in which deletions of *veA, velB*, and *laeA* have previously been shown to substantially increase transcription of the other members of the complex and, where examined, over-expression reduced expression of other gene members [3], [11]. However, consistent with the conidial phenotype of *llmF* mutants, the key asexual developmental activator *brlA* shows decreased expression in the Δ*llmF* strain and increased expression in the OE *llmF* strain (Figure 7).

### Cytoplasmic LlmF alters VeA localization in response to light

**Figure 8:**
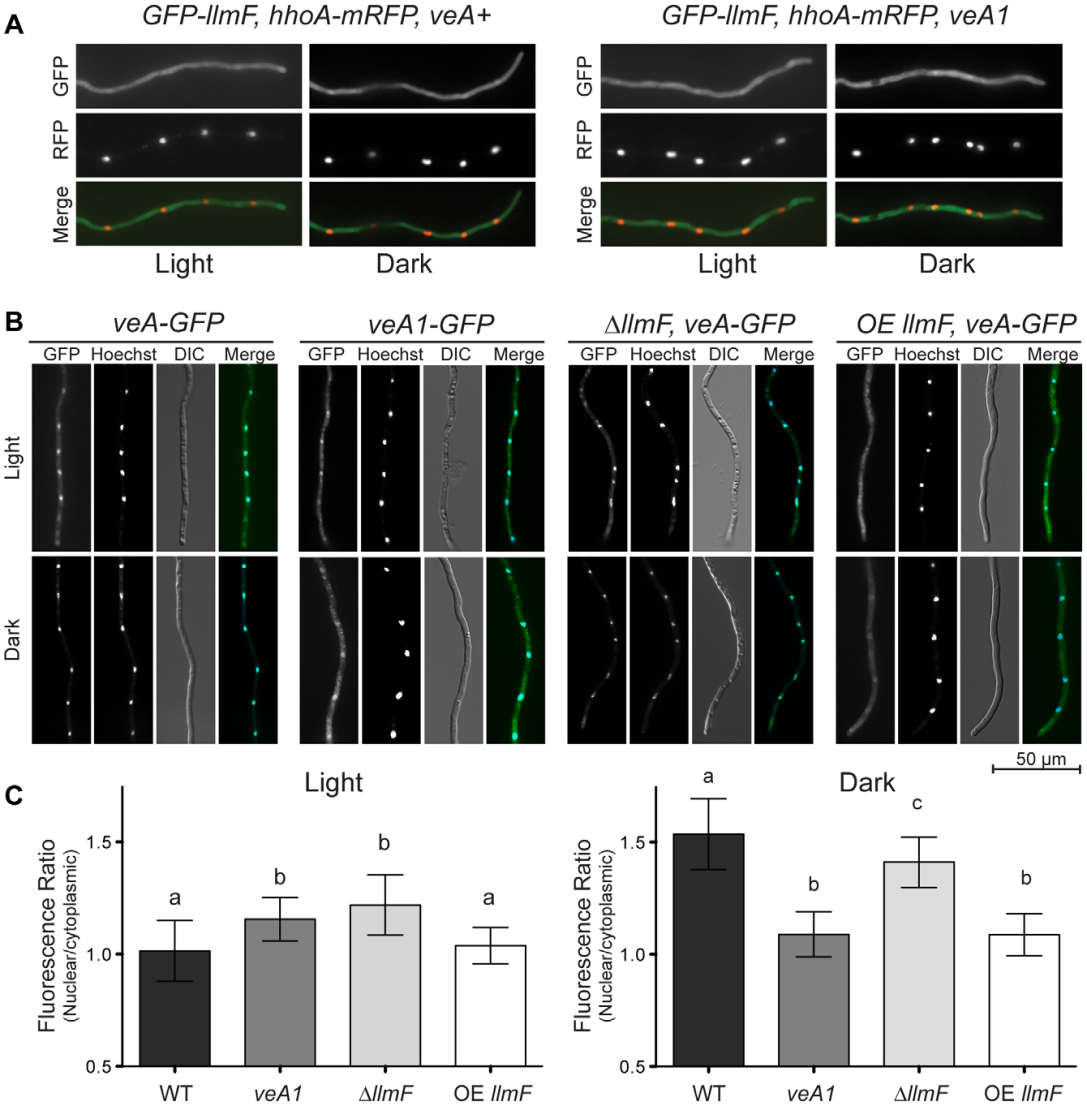
LlmF localizes to the cytoplasm and controls the subcellular localization of VeA. (A) The localization of LlmF was determined by constructing an N terminal GFP-LlmF fusion protein driven by the *gpdA* promoter. Germlings were grown on coverslips submerged in liquid minimal media overnight at 30°C. GFP fluorescence appears throughout the hyphae independent of light or the *veA* allele, as GFP-LlmF localization does not change in a *veA+* versus *veA1* background. Nuclei are shown with histone H1 labeled with mRFP (*hhoA-mRFP*). (B) VeA localization is determined by illumination. In the light VeA is found in the cytoplasm and the nucleus, while in the dark VeA concentrates in the nucleus. The truncated VeA1 protein is blind to light, as its localization does not change depending on illumination. In an OE *llmF* background, VeA does not accumulate in the nucleus when grown in the dark, while Δ*llmF* strains show similar localization patterns as wild type. (C) Quantification of VeA-GFP and VeA1-GFP fluorescence was done by measuring 50 nuclei and 50 cytoplasmic regions using Zeiss AxioVision Software 4.7 and statistical significance was calculated separately for light and dark conditions using an ANOVA and represented on the graph using lower case letters (a, b, and c). Error bars indicate standard deviation.

VeA has previously been shown to be a light regulated protein; in the light VeA is localized mainly in the cytoplasm, however in the dark VeA is almost exclusively nuclear [18]. On the other hand, LaeA is located in the nucleus independent of light but requires nuclear VeA for control of secondary metabolism and some aspects of sexuality [2], [3]. Since VeA is required for LaeA function, it seemed plausible that LlmF and LaeA could be competing for VeA binding, either directly or indirectly. Additionally, *llmF* mutants do not show phenotypes in a mutant *veA1* background suggesting that nucleo-cytoplasmic localization of VeA could be altered compared to wild type. To test this hypothesis, we first tagged LlmF with GFP at both the N and C terminus independently. By phenotypic analysis, C terminal LlmF-GFP constructs were nonfunctional; therefore we used the functional N terminal GFP-LlmF strains and determined LlmF was located primarily in the cytoplasm independent of light (Figure 8A). Additionally, the localization of GFP-LlmF in a *veA1* background was tested and displayed similar localization (Figure 8A).

While LaeA is constitutively nuclear and LlmF cytoplasmic, we reasoned that competition for VeA binding could be assessed by the subcellular localization of VeA. To test this hypothesis, VeA-GFP was visualized in Δ*llmF* and OE *llmF* genetic backgrounds. Interestingly, VeA-GFP was mis-localized in response to light conditions in both the Δ*llmF* and OE *llmF* backgrounds. Specifically, slightly more VeA accumulated in the nucleus under light conditions in a Δ*llmF* background compared to wild type whereas VeA failed to accumulate in the nucleus under dark conditions in an OE *llmF* background, phenocopying the VeA1-GFP localization pattern (Figure 8B and 8C). These results indicate that increased nuclear VeA could be responsible for the increased production of sterigmatocystin in Δ*llmF* strains and the cytoplasmic location of VeA in the OE *llmF* background the reason for decreased sterigmatocystin and sexual development in this strain.

### LlmF does not interact with VelB or the nuclear importin α

**Figure 9:**
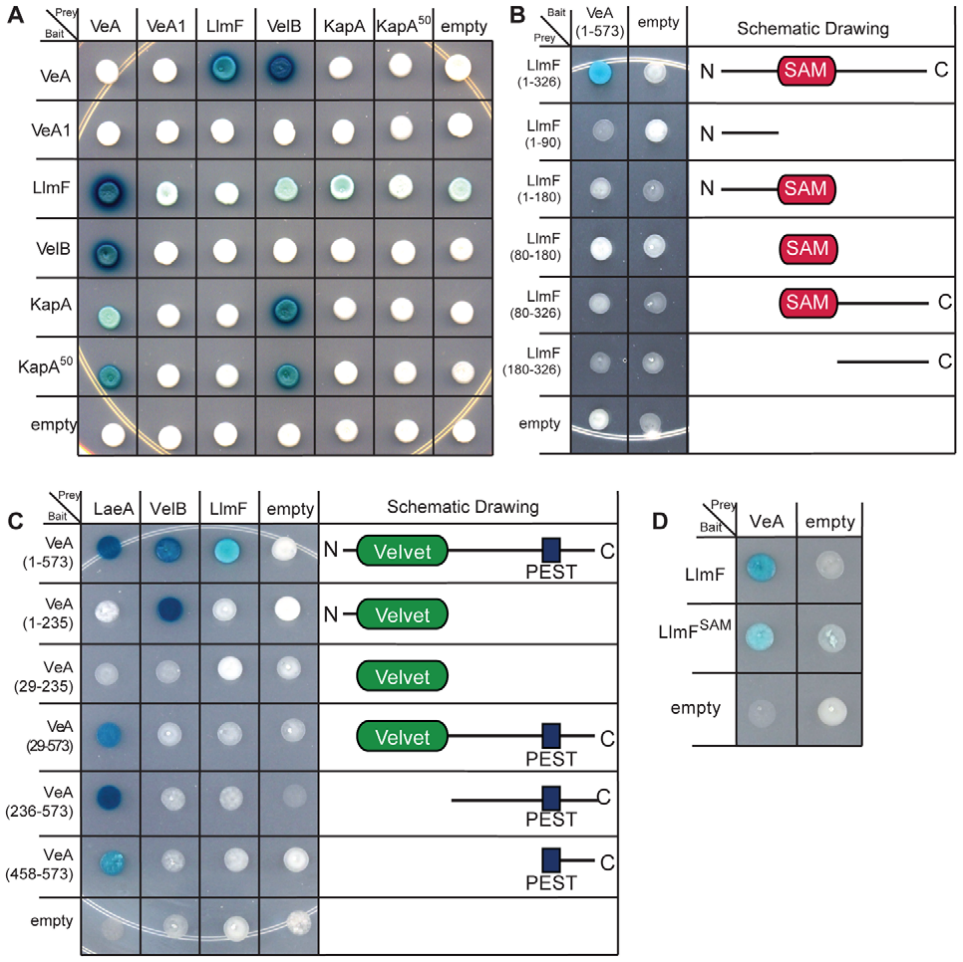
LlmF does not interact with VelB or KapA and only full-length LlmF interacts with VeA. (A) A directed yeast-two-hybrid analysis indicates that LlmF is not able to interact with VelB, full length KapA, or KapA^50^, which is missing the first 79 amino acids containing the importin β interaction domain. A positive interaction is indicated by a blue change in color via the *lacZ* reporter when grown on media containing X-gal. (B) Five truncation mutants of LlmF were constructed and tested against full length VeA in the yeast-two-hybrid assay. (C) Similarly, five different VeA truncations were created based on the locations of the predicted domain structure; the velvet domain is located from amino acid position 29–235 and the PEST domain is located from 458–573. The VeA truncations were tested in the yeast-two-hybrid assay against full length LaeA, VelB, and LlmF. (D) VeA is capable of interacting with LlmF^SAM^, which contains a mutation in motif I of the SAM binding site of LlmF.

Nuclear import of VeA in response to light is mediated by importin α (KapA) [18], [29]. Additionally, VelB is hypothesized to be a cytoplasmic protein that enters the nucleus through its interaction with VeA [3]. Hence, the model for VeA nuclear import is as follows: KapA recognizes and binds the nuclear localization signal of VeA and along with VelB, the KapA-VeA-VelB transient complex is transferred to the nuclear pore. To determine if LlmF could be altering VeA localization through disruption of VeA-VelB interaction or KapA-VeA interaction, we tested for the ability for LlmF to interact with either KapA or VelB. Figure 9A illustrates that neither KapA nor VelB are interaction partners for LlmF in the yeast-two-hybrid assay. These data also indicate that KapA is capable of recognizing VelB in the absence of VeA, however previous data suggests that VelB requires VeA for nuclear import [3].

### Full-length LlmF is required for interaction with VeA

Since LlmF interacted with only VeA and not VelB or KapA, we hypothesized that VeA-LlmF interaction could be interfering with KapA recognition and subsequent nuclear import. As previous data have indicated that KapA recognizes the VeA-VelB dimer for nuclear import [3], [29], to see if LlmF and VelB interacted with VeA in the same domains, we mapped the protein interaction site of VeA-LlmF using the directed yeast-two-hybrid assay. Testing the interaction of full length VeA with 5 different truncations of LlmF showed that only full length LlmF is capable of interacting with VeA in this assay (Figure 9B). Similarly, when 5 different truncations of VeA were tested as interaction partners with LlmF, similar results were obtained in which full length VeA is required for interaction with full length LlmF (Figure 9C).

Simultaneously, we were able to refine the interaction domains of VeA-LaeA and VeA-VelB. Previously Bayram et al. [3] showed that VelB interacts with the N terminus of VeA (1–300 aa) while LaeA interacts with the C terminus of VeA (276–573 aa). Here our data suggest that VelB interacts with the N terminus of VeA (1–235 aa), however the first 28 aa are necessary for this interaction as VelB did not interact with just the velvet domain of VeA (29–235) (Figure 9C). We were also able to show that LaeA can interact with the C terminal 115 amino acids of VeA (Figure 9C). Since our *in vivo* LlmF SAM binding site mutant was not functional (Figure 6), we tested for interaction between VeA and LlmF^SAM^, and found LlmF^SAM^ interacted with VeA (Figure 9D). Taken together, these data suggest that LlmF-VeA interaction is relatively weak in comparison to the heterotrimeric complex of VelB-VeA-LaeA.

### LlmF does not methylate VeA, VelB, or KapA *in vitro*

**Figure 10:**
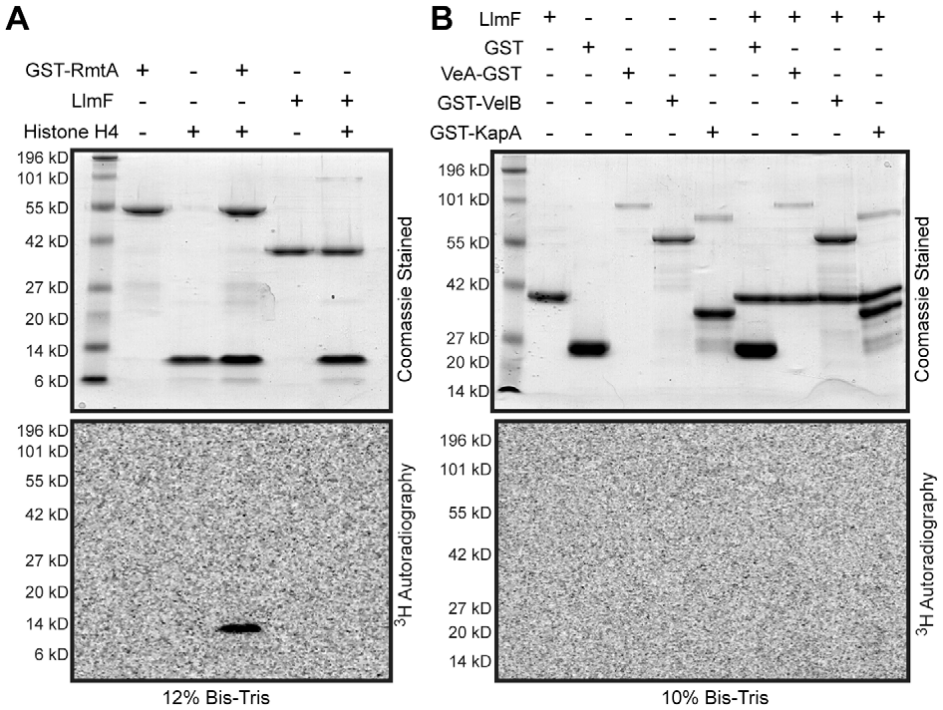
LlmF does not methylate the velvet complex *in vitro*. (A) An *in vitro* methylation assay was conducted by incubation of the recombinant proteins with ^3^H-SAM for 1 hour at 30°C. The reactions were stopped by addition of SDS sample buffer and electrophoresed on Bis-Tris SDS-PAGE gels. The gels were treated with fluorography enhancing solution (En^3^Hance, Perkin Elmer), dried, and exposed to a tritium phosphor screen for 2 weeks. GST-RmtA and human recombinant histone H4 (NEB) served as a positive control. Methylation of histone H4 by GST-RmtA was visualized via ^3^H autoradiography. (B) Using the same *in vitro* methylation assay, members of the velvet complex (VeA, VelB, and KapA) were tested as methylation substrates for LlmF. Under these conditions, LlmF was unable to methylate any of the proteins tested.

Since a mutation in the SAM binding site of LlmF did not disrupt its ability to interact with VeA (Figure 9D) but did render the protein inactive *in vivo* (Figure 6), we hypothesized that LlmF could be directly methylating VeA or other components involved in nuclear import of VeA, such as VelB and KapA. To test this hypothesis, recombinant LlmF was incubated with tritiated SAM (^3^H SAM) in the presence of GST, His_6_-VeA-GST-S-tag, GST-VelB, and GST-KapA and subsequently, methylation was measured by fluorography. As a positive control, GST-RmtA was incubated with its substrate of histone H4 as previously described [30]. These data indicated that *in vitro* LlmF does not methylate histone H4, VeA, VelB, or KapA (Figure 10A and 10B).

## Discussion

The multifaceted regulatory network that controls developmental differentiation and secondary metabolism in fungi is dependent on the velvet complex. Here we describe LlmF, a LaeA-like methyltransferase that constitutes a new interaction partner for VeA. LlmF is a negative regulator of sexual development and secondary metabolism through its control of VeA subcellular localization. These results provide strong evidence for a complex system regulating VeA subcellular compartmentalization as an important layer of control for development and secondary metabolism.

Using an *in silico* approach we identified several loci from the *A. nidulans* genome annotation that have sequence homology to LaeA (Figure 1), one of which (LlmF) was able to interact with VeA in a yeast-two-hybrid assay, an *in vitro* GST pull-down assay, and an *in vivo* pull-down experiment (Figure 4), thus identifying LlmF as a potential LaeA mimic or competitor. Deletion and overexpression (OE) LlmF mutants established that LlmF exhibited properties opposite of those of LaeA, specifically that LlmF repressed both sterigmatocystin and sexual development (Figure 3 and Figure 6). Interestingly, we also observed that in strains where *llmF* was overexpressed, sexual development was decreased in the dark, a phenotype reminiscent of the *veA1* allele. Using double mutants of Δ*llmF* with *veA1*, Δ*laeA*, and Δ*veA* it was apparent that LlmF control of secondary metabolism and development required LaeA and the full length VeA protein. Jiang et al. [13] also reported finding putative methyltransferases other than LaeA that were capable of interacting with VeA in *F. graminearum*, suggesting that LlmF may be conserved in other filamentous fungi.

**Figure 11:**
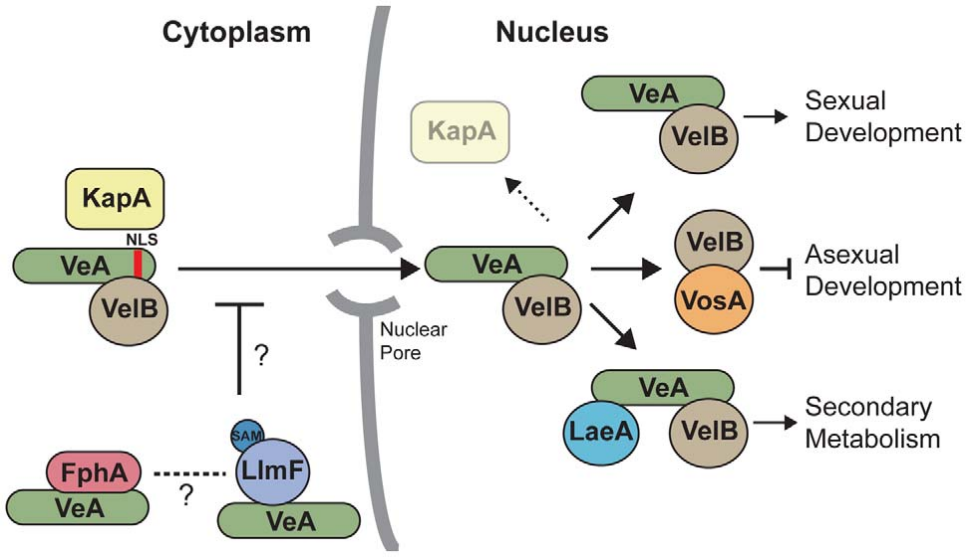
A model illustrates that LlmF controls VeA subcellular localization through methylation of an unknown substrate. Cytoplasmic VeA-VelB dimer is recognized by the importin α (KapA) and subsequently imported through the nuclear pore complex. After nuclear import, KapA dissociates where the VeA-VelB dimer functions to activate sexual development, VelB forms a dimer with VosA to repress asexual development, and the LaeA-VeA-VelB heterotrimeric complex forms that activates secondary metabolism. The data presented here depicts a role for a LlmF-VeA transient complex repressing the nuclear import of VeA primarily through the putative methylation activity of LlmF. The red light sensing phytochrome, FphA, also controls the subcellular localization of VeA [20], however it is unknown if FphA and LlmF share a pathway or independently regulate VeA subcellular localization.

Compartmentalization of proteins in eukaryotic cells is vital for proper growth and development. For example, several DNA binding transcription factors have been shown to shuttle between the cytoplasm and nucleus thereby directing gene transcription through subcellular localization [31]–[36] and proper localization of metabolic enzymes to organelles such as the peroxisome or mitochondria are critical for cellular function [37]–[39]. Here we observe the consequences of mis-localization of VeA mediated by LlmF for fungal development. Previous studies showed that VeA shuttles between the cytoplasm and nucleus in response to light [18]. VeA is hypothesized to act as a scaffold protein and mediate developmental pathways in response to illumination through regulation of its subcellular location [5], [23]. When the fungus is exposed to conditions favoring asexual development, *e.g.* light, VeA is located mainly in the cytoplasm. However, when there is no light, there is increased nuclear transport of the VeA-VelB heterodimer via the importin α (KapA), where they interact with the nuclear LaeA to induce sexual development and secondary metabolism (Figure 11). Our data implicate LlmF as a VeA cytoplasmic retention molecule contributing to proper location of VeA at the right developmental time point.

Deletion of the red light sensing phytochrome photoreceptor FphA results in increased VeA accumulation in the nucleus under light conditions, reminiscent of the Δ*llmF* background [20]. Blumenstein et al. [40] report that FphA is a cytoplasmic protein and functions to repress sexual development under red light. This would be consistent with the phenotypes observed with Δ*llmF*, however subsequent bimolecular fluorescence complementation (BiFC) assays suggest that FphA interacts with VeA in the nucleus [21]. It is also important to note that *In vivo* tandem affinity tag purification (TAP) of VeA resulted in co-purification of VelB, LaeA, and KapA [3], however did not pull-down LlmF or FphA. A plausible explanation for this phenomenon is that different culture conditions were used, most notably Bayram et al. [3] used sexual developmental culture conditions in the TAP purification of VeA. We suggest that these data indicate that LlmF (and potentially other proteins) may form a transient interaction with VeA in the cytoplasm thereby controlling its subcellular localization, while the stable LaeA-VeA-VelB complex forms in the nucleus.

Our data present evidence that cytoplasmic proteins interacting with VeA (LlmF and possibly FphA) control VeA localization, leading to downstream impacts on development and secondary metabolism. Here we present a model (Figure 11) where VeA retention by LlmF likely involves post-translational modifications precluding nuclear localization of VeA with a possible secondary effect of occlusion of KapA or VelB from cytoplasmic VeA. Post-translational modifications are known to both inhibit and enhance nuclear uptake depending on the cargo protein. For example, phosphorylation enhances the nuclear localization of the large tumor antigen of simian-virus 40 (SV40); conversely phosphorylation inhibits nuclear transport of Msn2p, a yeast transcriptional regulator of stress responses [31] and similarly inhibits nuclear transport of AflR [41]. Recently MpkB (Fus3) has been shown to be the kinase responsible for phosphorylating VeA, however VeA localization was not altered in the Δ*mpkB* background. [42]. More relevant to the putative methyltransferase ability of LlmF, methylation has also been linked to nuclear transport, as methylation of arginine residues in the NLS of RNA helicase A by the PRMT1 is required for transport into the nucleus [43].

To address if the putative methyltransferase ability of LlmF contributed to VeA localization, we constructed a SAM binding domain mutant of LlmF. Using the yeast-two-hybrid assay we determined that LlmF^SAM^ was still capable of binding to VeA, however was not functional *in vivo*. Although this does not preclude a role for LlmF in blocking KapA/VelB access to VeA, this result does suggest that the main role of LlmF in directing VeA localization involved a methylation activity. Thus we tested the ability of LlmF to methylate the known proteins involved in VeA nuclear import, which are VeA, VelB, and KapA. LlmF was unable to methylate any of these proteins in an *in vitro* methylation assay, which suggests another methylation substrate of LlmF is involved in VeA import.

Understanding the dynamics of the velvet complex cellular compartmentalization is crucial towards identifying regulatory networks of the fungal cell. Since VeA protein levels are constant over all developmental stages of the fungus, the subcellular localization of VeA plays a large role in proper response to environmental cues, *i.e*. development and secondary metabolism. It is not surprising then, that several mechanisms could exist to ensure proper localization of VeA such as mediation by LlmF. Future studies on proteins or small molecules that alter the cellular location of VeA could provide a novel form of controlling production of secondary metabolites, which has implications for reduction of contaminating toxins in food supplies as well as increasing production of beneficial metabolites for industrial applications.

## Materials and Methods

Fungal strains used in this study are listed in Table S1, plasmids used in this study are listed in Table S2, and primers used in this study are listed in Table S3. All strains were maintained on glucose minimal medium (GMM) [44] at 37°C and when appropriate were supplemented with 5 mM uracil, 5 mM uridine, 0.5 µM pyridoxine-HCl, 1 g L-tryptophan per liter, 0.125 g L-methionine per liter, and 1 µM p-aminobenzoate. Standard techniques were used for nucleic acid manipulations according to [45].

### *In silico* identification of putative methyltransferases in *A. nidulans*

Bioinformatic analysis of the *A. nidulans* genome was done using a combination of the Broad Institute Aspergillus Comparative database (http://www.broadinstitute.org/annotation/genome/aspergillus_group/) and the Aspergillus Genome Database [46]. SAM binding domain containing proteins were identified by retrieving all predicted proteins in the genome that contained an annotated methyltransferase domain from the *Aspergillus* Comparative database at the Broad Institute. The resulting 80 protein sequences were then manually culled and those that corresponded to polyketide synthases (15) or resided inside secondary metabolite gene clusters (9) were removed from the analysis. Clr4p from *Schizosaccharomyces pombe*, StcP from *A. flavus*, and 36 previously described methyltransferase from *Saccharomyces cerevisiae* were manually added from PubMed to the analysis. A ClustalW alignment and inferred phylogenetic tree was completed by the use of MegAlign Software (DNASTAR). Bootstrapping was performed using 1000 trials to determine tree branch lengths using MegAlign.

### Construction of mutant fungal strains

To assess the function of 8 LaeA-like methyltransferases (AN2165, AN8945, AN7933, AN5416, AN6749, AN5874, AN8833, and AN9193) gene disruption constructs were created to independently replace the putative ORFs with the *A. fumigatus pyrG* gene. Gene replacement constructs were created using fusion PCR [47]–[49]. Briefly, PCR products corresponding to approximately 1 kb upstream and downstream of the ORF were amplified from genomic DNA, subsequently gel purified, and then PCR-fused to flank either side of the *A. fumigatus pyrG* gene. The fusion PCR products were then used to transform RJMP1.49 [50] according to previously described procedures [51] with a minor modification of embedding protoplasts in molten 0.75% agar. Single gene replacement mutants were identified by PCR and subsequent Southern analysis (Figure S1 and data not shown). Prototrophic gene replacement strains as well as double mutants were constructed by sexual recombination (Table S1). Creation of VeA and VeA1 S-tagged strains were achieved via insertion of the C terminal S-tag construct from pAO81 [48] into RJMP1.27 and RJMP1.1. An *llmF* over-expression vector (pJMP22) was constructed by PCR fusion of the *gpdA* promoter to the ORF of *llmF* and subsequently an *EcoRI-NotI* fragment was cloned into the *pyroA* targeting vector pJW53 [52]. A SAM binding domain mutant (LlmF^G91A, G93A^) was constructed by quick-change mutagenesis (Stratagene) of pJMP22 to construct pJMP105. C-terminal and N-terminal *llmF* GFP fusions were constructed by insertion of GFP (amplified from pFNO3 and pSK505 respectively) into pJMP22 via PCR mediated insertion [53] to construct pJMP23 (OE *llmF*-GFP) and pJMP102 (OE GFP-GA_5_-*llmF*). The N terminal TAP tag was PCR amplified from pME2968 [54] and used to replace the GFP fragment in pJMP102 via PCR mediated insertion, yielding pJMP106 (OE TAP-GA_5_-*llmF*). Over-expression (OE) of *llmF*, GFP, *llmF^SAM^*, and TAP fusions was accomplished by transformation of pJMP22, pJMP23, pJMP102, pJMP105, and pJMP106 into RJMP1.59. Strains confirmed by Southern blot (data not shown) were subsequently crossed to prototrophy (Table S1).

### Fungal physiology experiments

Cultures for analysis of sporulation and production of secondary metabolites were set up by overlay inoculation of 1×10^6^ spores in molten GMM top agar containing 0.75% agar and subsequent incubation at 37°C. Three 10 mm cores were taken from each plate and homogenized in 3 mL of sterile water. Analysis of sterigmatocystin was done from cultures grown in constant light and constant dark for 4 days. Secondary metabolites were extracted with an equal volume of ethyl acetate and the organic layer was dried down. The dried extract was resuspended in 100 µL of ethyl acetate, 10 µL was spotted on silica backed thin layer chromatography plates (Whatman #4410-221), and separated using toluene: ethyl acetate: acetic acid (8∶1∶1) as a solvent. To visualize sterigmatocystin, the TLC plates were dried and sprayed with 15% aluminum chloride in ethanol and imaged under UV light (254 nm).

Sporulation was measured as previously described [24] by counting ascospores and conidia with a hemocytometer from sexual induced overlay-inoculated cultures that were grown for 5 days on GMM.

### Assaying protein–protein interactions: Yeast-two-hybrid

To determine if LlmF could physically interact with VeA, a directed yeast-two-hybrid system was used based on the LexA DNA binding domain [55], which has previously been used to map interaction sites of the VelB-VeA-LaeA complex [3]. Total RNA was extracted from WIM126 grown in both asexual and sexual developmental conditions, the RNA was pooled and cDNA was constructed. A PCR product was obtained using LlmF gene specific primers (Table S3) and subsequently cloned into pACT2 (Clonetech) and confirmed by sequencing. This cDNA was subsequently moved into the ‘bait’ vector (pTLexA) and the ‘prey’ vector (pGAD424). The appropriate plasmids were then transformed into the *Saccharomyces cerevisiae* L40 host [56] according to previously described protocols [57], and protein-protein interactions were measured using the histidine growth reporter as well as the *lacZ* X-gal colorimetric reporter.

### Recombinant protein purification and GST pull-downs

Heterologous expression and subsequent purification of proteins was conducted in *E. coli* BL21 (λDE3) using a combination of GST fusion pGEX (GE Healthcare) and N terminal 6X histidine - C terminal S-tag fusion vector pRSF-1b (EMD Biosciences). Briefly, GST fusions were constructed for LaeA, LlmF, VelB, and KapA which were purified from cell lysates using glutathione sepharose 6B (GE healthcare) following manufacturers recommendations. LlmF was cleaved from GST-LlmF using the Thrombin Cleavage Capture Kit (EMD Biosciences). An *E. coli* codon-optimized version of VeA was obtained from Reinhard Fischer, which was subsequently PCR amplified and cloned into pBluescript II SK- (pJMP70). An *EcoRI-XhoI* fragment from pJMP70 was sub-cloned into pRSF-1b (EMD Biosciences) that yielded a His_6_-VeA-S-tag fusion protein, which was purified using Ni-NTA resin (Qiagen) according to manufacturer's recommendations. A C terminal GST tag from pJMP89 was inserted into the *XhoI* site of pJMP126 to construct pJMP134 (His_6_-VeA-GST-S-tag), which was subsequently tandem purified with Ni-NTA resin followed by glutathione sepharose 6B (GE healthcare).

GST pull-down experiments were conducted essentially as previously described [45]. Briefly, 10 µg of purified GST tagged proteins (GST-LaeA, GST-VelB, GST-LlmF), 25 µL of glutathione sepharose 4B (GE healthcare), and 50 µL of purified His_6_-VeA-S-tag were co-incubated for 3 hours at 4°C in PBS buffer (140 mM NaCl, 2.7 mM KCl, 10 mM Na_2_HPO_4_, 1.8 mM KH_2_PO_4_, 1 mM DTT, pH 7.3). The resin was subsequently washed 4 times with PBS buffer, boiled for 10 minutes in 2X NuPage LDS sample buffer (Invitrogen), electrophoresed on a 9% Bis-Tris polyacrylamide gel, transferred to nitrocellulose membrane, and stained with 0.1% Ponceau S solution (Sigma). Immunodetection of His_6_-VeA-S-tag was performed using a Rabbit α-S-tag antibody at 1∶2,000 dilution (Immunology Consultants Laboratory, Inc) followed by Goat anti-Rabbit-HRP 1∶15,000 dilution (Pierce) and chemiluminescence detection with Pierce West Pico Substrate (Pierce).

### *In vivo* TAP pull-down experiments

One-liter liquid shaking cultures of GMM were inoculated with 5×10^5^ spores/mL and grown for 30°C at 230 RPM for 36 hours. Mycelia was harvested by filtration through miracloth (EMD Biosciences), frozen in liquid nitrogen, and ground via cryo-compaction using a mixer mill (Retsch MM 400). Tandem affinity purification was conducted similarly as previously described [54] with a few minor modifications. Ground mycelia were extracted in buffer B250 (100 mM Tris-HCl, 250 mM NaCl, 10% glycerol, 1 mM EDTA, 1 mM DTT, 1 mM PMSF, 0.05% Tergitol-NP40, pH 7.5, and Roche Complete Protease inhibitors) and crude lysate was obtained by centrifugation at 47,810 *x g* for 30 minutes. The clarified crude lysate was incubated with 350 µL of IgG Sepharose 6 Fast Flow (GE Healthcare) for 3 hours on a rocking platform at 4°C and subsequently loaded into a 10 mL chromatography column (BioRad Poly-Prep). The IgG Sepharose was washed twice with 10 mL of buffer W250 (40 mM Tris-HCl, 250 mM NaCl, 1 mM PMSF, 1 mM DTT, 0.1% Tergitol-NP40, pH 8.0), once with 10 mL of buffer W150 (40 mM Tris-HCl, 150 mM NaCl, 1 mM PMSF, 1 mM DTT, 0.1% Tergitol-NP40, pH 8.0), and once with 10 mL of buffer TCB (40 mM Tris-HCl, 150 mM NaCl, 0.5 mM EDTA, 1 mM DTT, 0.1% Tergitol-NP40, pH 8.0). TEV cleavage was done by incubating the washed IgG Sepharose in 1 mL of TCB with 80 µL of rTEV (kind gift from Ivan Rayment, UW-Madison) overnight at 4°C on a rocking platform. The eluate was mixed with 500 µL of equilibrated calmodulin sepharose 4B (GE Healthcare) in 6 mL of CBB (40 mM Tris-HCl, 150 mM NaCl, 1 mM magnesium acetate, 1 mM imidazole, 2 mM CaCl_2_, 10 mM 2-mercaptoethanol, 0.1% Tergitol-NP40, pH 8.0) and incubated for 1 hour at 4°C on a rocking platform. The calmodulin sepharose was subsequently washed three times with 1 mL of CBB with 0.02% Tergitol-NP40 and finally eluted twice with 500 µL of CEB (40 mM Tris-HCl, 150 mM NaCl, 1 mM magnesium acetate, 1 mM imidazole, 20 mM EGTA, 10 mM 2-mercaptoethanol, pH 8.0). The eluate was TCA precipitated, resuspended in NuPAGE LDS sample buffer (Invitrogen), electrophoresed in a 10% Bis-Tris polyacrylamide gel, and transferred to Immobilon-P PVDF membrane (Millipore). Immunodetection of VeA and VeA1-S-tag was accomplished using Rabbit-α-S-tag at a 1∶2,000 dilution followed by Goat-α-Rabbit-Cy5 (GE Healthcare ECL Plex) at a 1∶2,000 dilution and imaged using a Typhoon FLA9000 fluorescent imager. The blot was stripped in 62.5 mM Tris-HCl, pH 6.5, 2% SDS, 100 mM 2-mercaptoethanol at 50°C for 30 minutes and washed twice in TBS (50 mM Tris-HCl, 150 mM NaCl, pH 7.4). The blot was then re-probed for detection of TAP-LlmF with Rabbit-α-calmodulin (Millipore) at a 1∶1,000 dilution and detected with the ECL Plex system as described above.

### *S*-adenosyl methionine binding assay

The ability of recombinant LaeA and LlmF to bind the methyl group donor *S*-adenosyl methionine (SAM) was determined through an *in vitro* ultraviolet-light (UV) crosslinking assay as previously described [58]. Briefly, 5 µg of recombinantly purified GST, GST-LaeA, GST-LlmF, and LlmF were incubated with 6 µL of 1.0 µCi/mL of ^3^H-SAM (Perkin Elmer) at room temperature for 20 minutes followed by irradiation with UV light on ice for an additional 30 minutes. To demonstrate active site binding, the competitive inhibitor *S*-adenosyl-homocysteine (SAH) was used at 1 µM. The reactions were stopped by addition of SDS-PAGE sample buffer and electrophoresed on a 10% Tricine-SDS-PAGE gel [59]. Proteins were transferred to a nitrocellulose membrane (Protran) and bound ^3^H-SAM was measured by 48-hour exposure to a tritium phosphor storage screen (GE Healthcare) following manufacturers recommendations.

### Microscopy

Localization of proteins was visualized with a C terminal GFP fusion of VeA [18] and N terminal GFP fusion of LlmF driven by the *gpdA* promoter. Cells were prepared as previously described with minor modifications [60]. Briefly, approximately 5×10^4^ conidia were inoculated in liquid GMM medium over sterile cover slips in petri dishes at 30°C overnight. Cover slips containing adherent germlings were transferred to glass slides with 1 µL of Hoechst 33258 (1 mg/mL) and imaged with a Zeiss AxioImager A10 equipped with a Zeiss EC Plan-NEOFLUAR 40X/1.3 Oil DIC objective, a series 120 X-Cite light source (EXFO), Zeiss filter set 10 for GFP, Zeiss filter set 00 for mRFP, and Zeiss filter set 49 for Hoechst. Microscope settings were identical for all samples and GFP quantification was achieved using the Zeiss AxioVision 4.7 software of 50 nuclei and 50 cytoplasmic areas for each strain.

### Northern analysis

Total RNA was extracted using TriZol (Invitrogen) using manufacturers recommendations. Detection of *llm* transcripts was achieved by analyzing mRNA from wild-type *A. nidulans* (RDIT9.32) grown under several different developmental conditions (Figure 2). Mycelia from sexual developmental induction cultures was scraped from the surface of solid media plate with a glass slide, lyophilized overnight, and subsequently total RNA was extracted using TriZol (Figure 6D). Vegetative growth was conducted by inoculation of 1×10^6^ spores/mL in 50 mL of liquid GMM at 37°C and 250 rpm. Asexual and sexual development was induced by transfer of mycelia grown for 24 hours in liquid shake to solid GMM plates and further incubated in either constant light (asexual induction) or constant dark (sexual development) at 37°C. Expression of velvet complex members was assayed from mycelia grown under asexual developmental conditions for 24 hours after transfer to solid media and sexual developmental conditions for 48 hours after transfer to solid media (Figure 7). ^32^P-labeled probes were made using standard techniques [45] and primers are listed in Table S3.

### *In vitro* methylation assay

Methylation assays were conducted essentially as previously described [30], with the following minor modifications. Five micrograms of purified LlmF was incubated with 5 µL of 1.0 µCi/mL of ^3^H-SAM (Perkin Elmer) and 5 µg of putative substrate protein in 1X methyltransferase buffer (50 mM HEPES, 100 mM NaCl, 2 mM DTT, pH 7.5) for 1 hour at 30°C. The reactions were stopped by addition of 4X sample buffer and the entire reactions were electrophoresed via Bis-Tris SDS-PAGE. Gels were subsequently stained with Coomassie Brilliant Blue R-250, impregnated with En^3^Hance fluorography enhancing solution (Perkin Elmer), dried, and exposed to a tritium phosphor storage screen (GE Healthcare) for 2 weeks.

## Supporting Information

Figure S1 Disruption of LaeA-like methyltransferases in *A. nidulans* was done using double-joint-PCR and double homologous recombination. A schematic (drawn to scale) and restriction enzyme sites illustrate the strategy taken to replace *llmF* (AN6749) with the *A. fumigatus pyrG* gene. Southern blots hybridized with a radiolabeled probe of the knockout cassette confirm that TJMP9.2, TJMP9.4, and TJMP9.5 harbor simple gene replacements. Restriction digests of genomic DNA using *BamHI + SacI* show the expected banding pattern: WT = 5.5 kb and 1.4 kb, Δ*llmF* = 4.2 kb, 1.4 kb, 1.1 kb, and 0.8 kb. Restriction digests using *EcoRV*: WT = 2.1 kb and 1.4 kb, ΔllmF = 5.3 kb.
(TIF)

Figure S2 Secondary metabolite analysis of LaeA-like methyltransferase deletion mutants reveals minor phenotypes. Simple gene replacements were constructed for all of the putative LaeA-like methyltransferases. The single deletion mutants were grown for 4 days by overlay inoculation on minimal media according to materials and methods. Secondary metabolites were extracted and analyzed via thin layer chromatography. TLC plates were sprayed with 15% aluminum chloride and visualized under UV light (254 nm). There are minor changes in production of sterigmatocystin in some of the strains grown under constant light, notably Δ*llmC* and Δ*llmJ* make slightly more sterigmatocystin than wild type. Strains used in this experiment are as follows: WT = RJMP103.5, ΔllmA = RJMP104.5, ΔllmB = RJMP112.39, ΔllmC = RJMP196.1, ΔllmD = RJMP107.8, ΔllmG = RJMP195.1, ΔllmI = RJMP111.3, ΔllmJ = RJMP113.3.
(TIF)

Figure S3 The cDNA of *llmF* contains 6 exons and encodes for a predicted SAM binding domain protein. (A) A cDNA corresponding to *llmF* was amplified and cloned from pooled mRNA isolated from WIM126 grown in both asexual and sexual development conditions. Sequencing of the cloned cDNA revealed 6 exons and a length of 981 bp. This differed slightly from the predicted mRNA in the annotated genome as the cDNA contained an additional exon. (B) The conserved domain database predicts that AN6749 encodes a putative s-adenosyl methionine (SAM) binding site.
(TIF)

Table S1 Strains of *Aspergillus nidulans* used in this study.
(DOCX)

Table S2 Plasmids and yeast strains used in this study.
(DOCX)

Table S3 Oligonucleotides used in this study.
(DOCX)
