# Supplementary figures and images for: Secondary Metabolism and Development Is Mediated by LlmF Control of VeA Subcellular Localization in Aspergillus nidulans
Source: PLoS Genet. 2013 Jan 17;9(1):e1003193. doi: 10.1371/journal.pgen.1003193 (PMC3547832; doi:10.1371/journal.pgen.1003193)

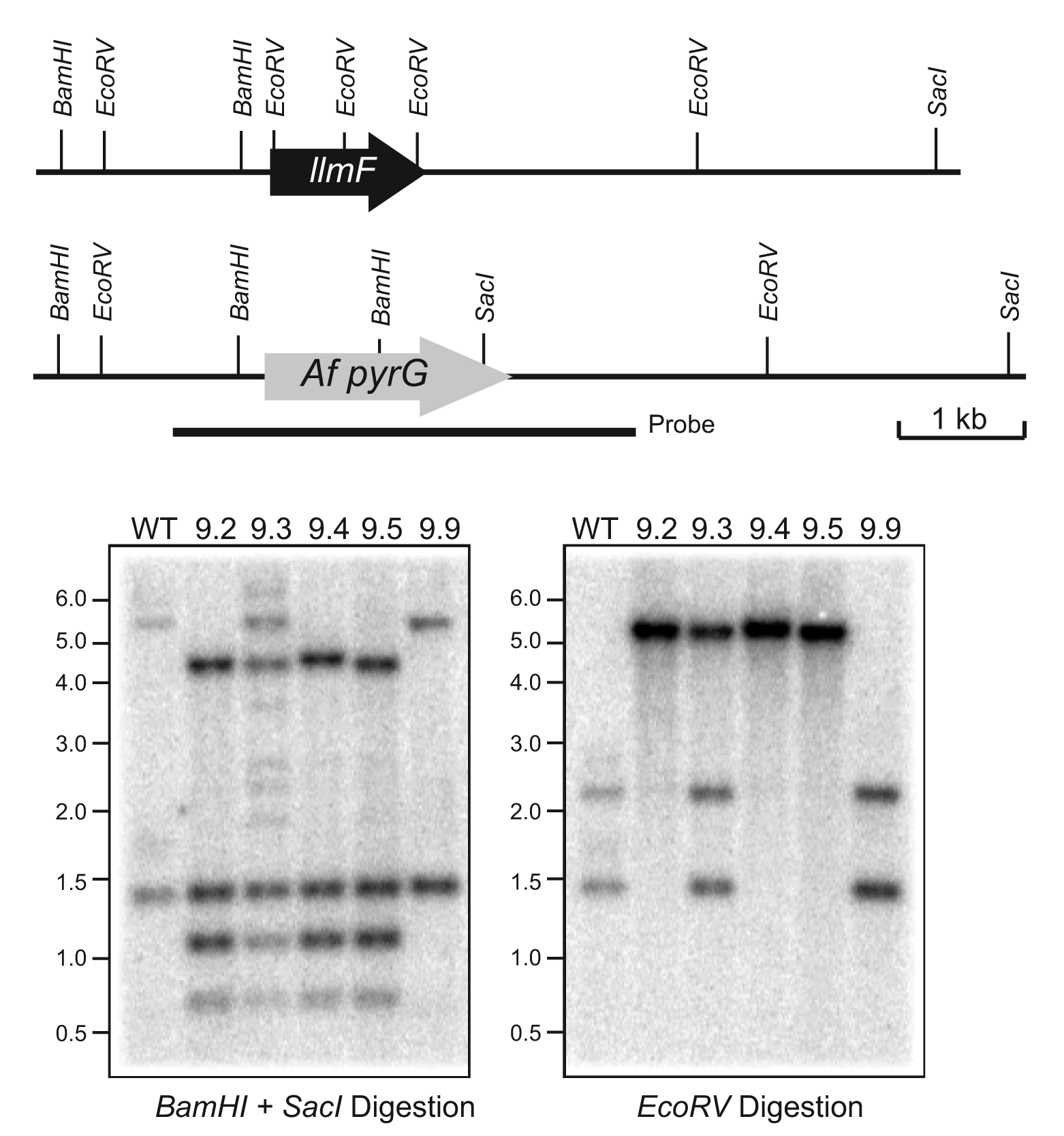

Supplement: Figure S1 — Disruption of LaeA-like methyltransferases in A. nidulans was done using double-joint-PCR and double homologous recombination. A schematic (drawn to scale) and restriction enzyme sites illustrate the strategy taken to replace llmF (AN6749) with the A. fumigatus pyrG gene. Southern blots hybridized with a radiolabeled probe of the knockout cassette confirm that TJMP9.2, TJMP9.4, and TJMP9.5 harbor simple gene replacements. Restriction digests of genomic DNA using BamHI + SacI show the expected banding pattern: WT = 5.5 kb and 1.4 kb, ΔllmF = 4.2 kb, 1.4 kb, 1.1 kb, and 0.8 kb. Restriction digests using EcoRV: WT = 2.1 kb and 1.4 kb, ΔllmF = 5.3 kb. (TIF) [file pgen.1003193.s001.tif]

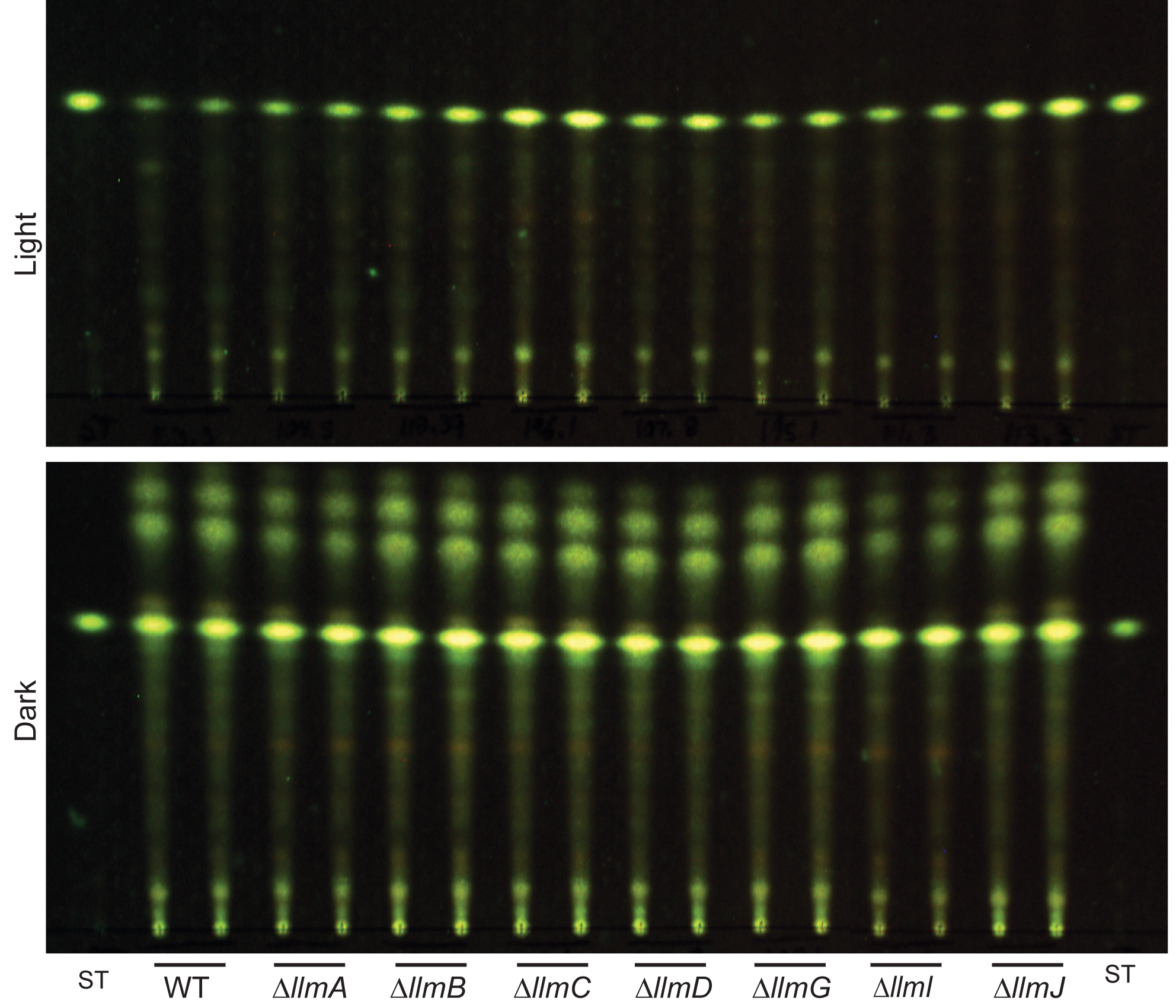

Supplement: Figure S2 — Secondary metabolite analysis of LaeA-like methyltransferase deletion mutants reveals minor phenotypes. Simple gene replacements were constructed for all of the putative LaeA-like methyltransferases. The single deletion mutants were grown for 4 days by overlay inoculation on minimal media according to materials and methods. Secondary metabolites were extracted and analyzed via thin layer chromatography. TLC plates were sprayed with 15% aluminum chloride and visualized under UV light (254 nm). There are minor changes in production of sterigmatocystin in some of the strains grown under constant light, notably ΔllmC and ΔllmJ make slightly more sterigmatocystin than wild type. Strains used in this experiment are as follows: WT = RJMP103.5, ΔllmA = RJMP104.5, ΔllmB = RJMP112.39, ΔllmC = RJMP196.1, ΔllmD = RJMP107.8, ΔllmG = RJMP195.1, ΔllmI = RJMP111.3, ΔllmJ = RJMP113.3. (TIF) [file pgen.1003193.s002.tif]

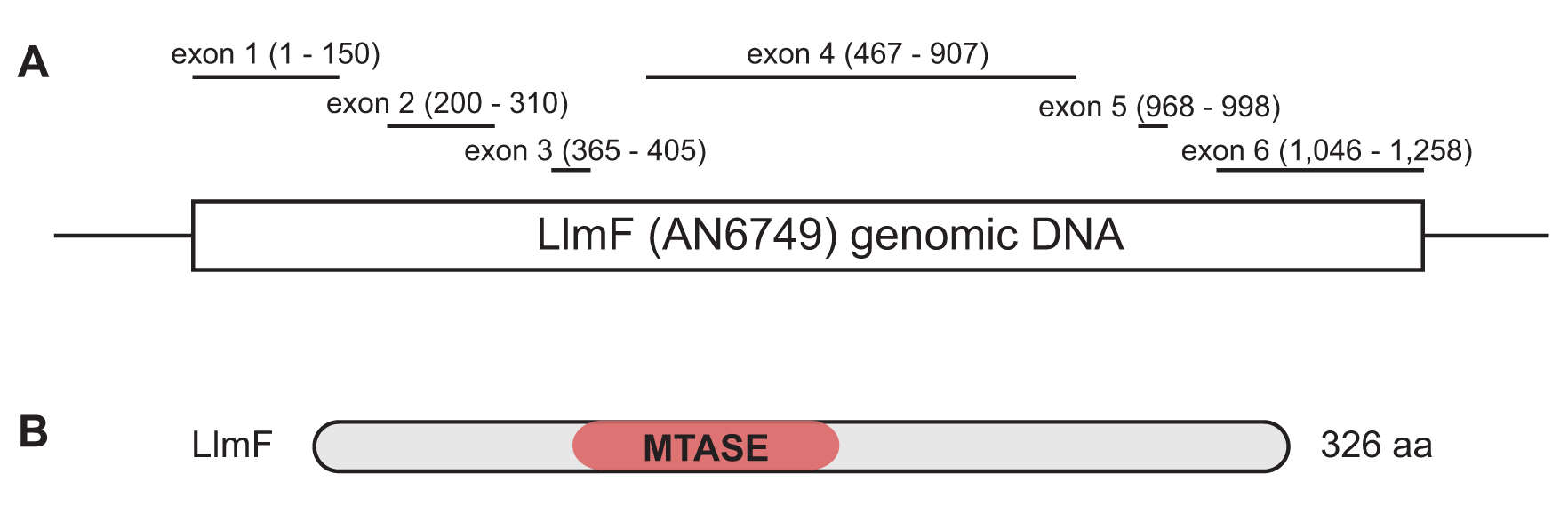

Supplement: Figure S3 — The cDNA of llmF contains 6 exons and encodes for a predicted SAM binding domain protein. (A) A cDNA corresponding to llmF was amplified and cloned from pooled mRNA isolated from WIM126 grown in both asexual and sexual development conditions. Sequencing of the cloned cDNA revealed 6 exons and a length of 981 bp. This differed slightly from the predicted mRNA in the annotated genome as the cDNA contained an additional exon. (B) The conserved domain database predicts that AN6749 encodes a putative s-adenosyl methionine (SAM) binding site. (TIF) [file pgen.1003193.s003.tif]
