# Supplementary material for: Secondary Metabolism and Development Is Mediated by LlmF Control of VeA Subcellular Localization in Aspergillus nidulans
Source: PLoS Genet. 2013 Jan 17;9(1):e1003193. doi: 10.1371/journal.pgen.1003193 (PMC3547832; doi:10.1371/journal.pgen.1003193)
Supplement: Table S1 — Strains of Aspergillus nidulans used in this study. (DOCX) [file pgen.1003193.s004.docx]

Table S1. Strains of *Aspergillus nidulans* used in this study.

| **Strain** | **Genotype** | **Source** |
| --- | --- | --- |
| RDIT9.32 | Wild-type | [[52](#_ENREF_52)] |
| WIM126 | *yA2, pabaA1* | FGSC |
| RJMP103.5 | Wild-type | This study |
| RJW41A | *∆laeA::metG1* | [[3](#_ENREF_3)] |
| RJMP162.8 | *veA1* | This study |
| RJW112.2 | *∆veA::argB* | [[3](#_ENREF_3)] |
| RJMP1.49 | *pyrG89, pyroA4, ∆nkuA::argB* | [[50](#_ENREF_50)] |
| RJMP1.59 | *pyrG89, pyroA4* | [[24](#_ENREF_24)] |
| RJMP1.27 | *pyrG89, pyroA4, riboB2, ∆nkuA::argB* | This study |
| RJMP1.1 | *pyrG89, pyroA4, riboB2, ∆nkuA::argB, veA1* | This study |
| TJMP4.4 | *pyrG89, pyroA4, ∆llmA(AN2165)::Af-pyrG, ∆nkuA::argB* | This study |
| TJMP12.1 | *pyrG89, pyroA4, ∆llmB(AN8945)::Af-pyrG, ∆nkuA::argB* | This study |
| TJMP10.2 | *pyrG89, pyroA4, ∆llmC(AN7933)::Af-pyrG, ∆nkuA::argB* | This study |
| TJMP7.5 | *pyrG89, pyroA4, ∆llmD(AN5416)::Af-pyrG, ∆nkuA::argB* | This study |
| TJMP9.2 | *pyrG89, pyroA4, ∆llmF(AN6749)::Af-pyrG, ∆nkuA::argB* | This study |
| TJMP8.4 | *pyrG89, pyroA4, ∆llmG(AN5874)::Af-pyrG, ∆nkuA::argB* | This study |
| TJMP11.6 | *pyrG89, pyroA4, ∆llmI(AN8833)::Af-pyrG, ∆nkuA::argB* | This study |
| TJMP13.2 | *pyrG89, pyroA4, ∆llmJ(AN9193)::Af-pyrG, ∆nkuA::argB* | This study |
| TJMP57.10 | *pyrG89, pyroA4, riboB2, ∆nkuA::argB, veA::Stag::AfpyrG* | This study |
| TJMP58.9 | *pyrG89, pyroA4, riboB2, ∆nkuA:;argB, veA1::Stag::AfpyrG* | This study |
| TJMP49.9 | *pyrG89, pyroA::gpdA(p)::llmF* | This study |
| TJMP50.8 | *pyrG89, pyroA::gpdA(p)::llmF::GFP* | This study |
| TJMP104.20 | *pyrG89, pyroA::gpdA(p)::GFP::llmF* | This study |
| TJMP105.29 | *pyrG89, pyroA::gpdA(p)::llmF ^G91A,G93A^* | This study |
| TJMP106.7 | *pyrG89, pyroA::gpdA(p)::TAP::llmF* | This study |
| LO1353 | *pabaA1, pyrG89, hhoA::mRFP::Af-pyrG, wA3, yA2, veA1* | Berl Oakley |
| RJMP249.1 | *pyrG89, pyroA::gpdA(p)::TAP::llmF, veA::Stag::AfpyrG* | This study |
| RJMP250.2 | *pyrG89, pyroA::gpdA(p)::TAP::llmF, veA1::Stag::AfpyrG* | This study |
| RJMP101.17 | *pyrG89, ∆laeA::metG1, veA1* | This study |
| T17 | *pyroA4, veA::GFP::Af-pyrG* | [[18](#_ENREF_18)] |
| T13 | *pyroA4, veA1::GFP::Af-pyrG, wA3* | [[18](#_ENREF_18)] |
| RJMP104.5 | *pyrG89, ∆llmA::Af-pyrG* | This study |
| RJMP112.39 | *pyrG89, ∆llmB::Af-pyrG* | This study |
| RJMP196.1 | *pyrG89, ∆llmC::Af-pyrG* | This study |
| RJMP107.8 | *pyrG89, ∆llmD::Af-pyrG* | This study |
| RJMP109.26 | *pyrG89, ∆llmF::Af-pyrG* | This study |
| RJMP195.1 | *pyrG89, ∆llmG::Af-pyrG* | This study |
| RJMP111.3 | *pyrG89, ∆llmI::Af-pyrG* | This study |
| RJMP113.3 | *pyrG89, ∆llmJ::Af-pyrG* | This study |
| RJMP109.12 | *pyrG89, ∆llmF::Af-pyrG, veA1* | This study |
| RJMP109.10 | *pyrG89, ∆llmF::Af-pyrG, ∆laeA::metG1* | This study |
| RJMP119.3 | *pyrG89, ∆llmF::Af-pyrG, ∆veA::argB* | This study |
| RDIT30.34 | *metG1, trpC801, pyrG89, veA1* | [[17](#_ENREF_17)] |
| RJMP137.24 | *pyrG89, pyroA4, ∆llmF::Af-pyrG* | This study |
| RJMP136.3 | *pyrG89, ∆llmF::Af-pyrG, pyroA::gpdA(p)::llmF* | This study |
| RJMP136.6 | *pyrG89, ∆llmF::Af-pyrG, pyroA::gpdA(p)::llmF* | This study |
| RJMP120.5 | *pyrG89, ∆llmF::Af-pyrG, pyroA::gpdA(p)::llmF::GFP* | This study |
| RJMP205.5 | *hhoA::mRFP::Af-pyrG, pyroA::gpdA(p)::GFP::llmF* | This study |
| RJMP205.10 | *hhoA::mRFP::Af-pyrG, pyroA::gpdA(p)::GFP::llmF, veA1* | This study |
| RJMP207.1 | *pyrG89, ∆llmF::Af-pyrG, pyroA::gpdA(p)::llmF ^G91A,G93A^* | This study |
| RJMP144.6 | *veA::GFP::Af-pyrG* | This study |
| RJMP153.1 | *veA1::GFP::Af-pyrG, wA3* | This study |
| RJMP153.7 | *∆laeA::metG, veA::GFP::Af-pyrG* | This study |
| RJMP144.9 | *∆llmF::Af-pyrG, veA::GFP::Af-pyrG* | This study |
| RJMP143.5 | *pyroA::gpdA(p)::llmF, veA::GFP-Af-pyrG* | This study |
