# Supplementary material for: Secondary Metabolism and Development Is Mediated by LlmF Control of VeA Subcellular Localization in Aspergillus nidulans
Source: PLoS Genet. 2013 Jan 17;9(1):e1003193. doi: 10.1371/journal.pgen.1003193 (PMC3547832; doi:10.1371/journal.pgen.1003193)
Supplement: Table S2 — Plasmids and yeast strains used in this study. (DOCX) [file pgen.1003193.s005.docx]

Table S2. Plasmids and yeast strains used in this study

| **Plasmid/Strain** | **Description/Genotype** | **Source** |
| --- | --- | --- |
| L40 | *MATa trp1 leu2 his3 LYS2::lexA-HIS3 URA3::lexA-lacZ* | [[56](#_ENREF_56)] |
| pJW53 | ¾ pyroA in *SspI* site of pBluescript II SK- | [[52](#_ENREF_52)] |
| pJMP22 | *gpdA(p)::llmF* in pJW53 | This study |
| pJMP23 | *gpdA(p)::llmF::GA_4_::GFP* in pJW53 | This study |
| pJMP102 | *gpdA(p)::GFP::GA_4_::llmF* in pJW53 | This study |
| pJMP105 | *gpdA(p)::llmF^G91A, G93A^* in pJW53 | This study |
| pJMP106 | *gpdA(p)::TAP::GA_4_::llmF* in pJW53 | This study |
| pME2968 | N terminal TAP tag | [[54](#_ENREF_54)] |
| pAO81 | *GA_4_::Stag::Af-pyrG* | [[48](#_ENREF_48)] |
| pFNO3 | *GA_5_::GFP::Af-pyrG* | [[48](#_ENREF_48)] |
| pSK505 | *ptrA::gpdA(p)::GFP2-5::GA_4_::Af histone H2A* | [[62](#_ENREF_62)] |
| pACT2 | Gal4 BD vector | Clontech |
| pACT2-llmF | *llmF* cDNA in pACT2 | This study |
| pGEX4T-1 | GST fusion expression plasmid | GE Healthcare |
| pGEX4T-3 | GST fusion expression plasmid | GE Healthcare |
| pJMP32.4 | *llmF* cDNA in pGEX4T-1 | This study |
| pRMP15 | *laeA* cDNA in pGEX4T-1 | RM Perrin and NP Keller |
| pGST-velB | *velB* cDNA in pGEX4T-3 | This study |
| pJMP91 | *kapA* cDNA in pGEX4T-3 | This study |
| pET-veA | His_6_-T7-*veA* cDNA in pET28a (codon optimized) | Reinhard Fischer |
| pJMP70 | *veA* cDNA in pBluescript II SK- | This study |
| pRSF-1b | N terminal 6X His, C terminal S-tag | EMD Biochemical |
| pJMP126 | His_6_-*veA*-Stag in pRSF-1b | This study |
| pJMP89 | TEV-GST in pBluescript II SK | This study |
| pJMP134 | His_6_-*veA*-GST-Stag in pRSF-1b | This study |
| pTlexA | Y2H bait vector – LexA binding domain fusion, TRP1 | [[55](#_ENREF_55)] |
| pGAD424 | Y2H prey vector – GAL4 activation domain fusion, LEU2 | Clontech |
| pJMP48 | *llmF* cDNA in pTlexA | This study |
| pJMP49 | *llmF* cDNA in pGAD424 | This study |
| pNJ04 | *veA* cDNA in pTlexA | [[3](#_ENREF_3)] |
| pNJ05 | *veA* cDNA in pGAD424 | [[3](#_ENREF_3)] |
| pNJ08 | *veA1* cDNA in pTlexA | [[3](#_ENREF_3)] |
| pNJ09 | *veA1* cDNA in pGAD424 | [[3](#_ENREF_3)] |
| pNJ14 | *laeA* cDNA in pTlexA | [[3](#_ENREF_3)] |
| pNJ17 | *laeA* cDNA in pGAD424 | [[3](#_ENREF_3)] |
| pNJ20 | *velB* cDNA in pTlexA | [[3](#_ENREF_3)] |
| pNJ23 | *velB* cDNA in pGAD424 | [[3](#_ENREF_3)] |
| pJMP96 | *kapA* cDNA in pTlexA | This study |
| pJMP97 | *kapA* cDNA in pGAD424 | This study |
| pJMP103 | *kapA^∆1-79^* cDNA in pTlexA | This study |
| pJMP104 | *kapA^∆1-79^* cDNA in pGAD424 | This study |
| pJMP159 | *llmF^G91A,G93A^ cDNA* in pTLexA | This study |
| pJMP162 | *llmF (1-90aa)* cDNA in pGAD424 | This study |
| pJMP163 | *llmF (80-180aa)* cDNA in pGAD424 | This study |
| pJMP164 | *llmF (180-326aa)* cDNA in pGAD424 | This study |
| pJMP165 | *llmF (1-180aa)* cDNA in pGAD424 | This study |
| pJMP166 | *llmF (80-326aa)* cDNA in pGAD424 | This study |
| pJMP168 | *veA (1-235aa)* cDNA in pTLexA | This study |
| pJMP169 | *veA (29-235aa)* cDNA in pTLexA | This study |
| pJMP170 | *veA (129-573aa)* cDNA in pTLexA | This study |
| pJMP171 | *veA (236-573aa)* cDNA in pTLexA | This study |
| pJMP172 | *veA (458-573aa)* cDNA in pTLexA | This study |
