# Supplementary material for: Secondary Metabolism and Development Is Mediated by LlmF Control of VeA Subcellular Localization in Aspergillus nidulans
Source: PLoS Genet. 2013 Jan 17;9(1):e1003193. doi: 10.1371/journal.pgen.1003193 (PMC3547832; doi:10.1371/journal.pgen.1003193)
Supplement: Table S3 — Oligonucleotides used in this study. (DOCX) [file pgen.1003193.s006.docx]

Table S3. Oligonucleotides used in this study.

| **Primers** | **Sequence (5’ to 3’): restriction sites underlined** | **Purpose** |
| --- | --- | --- |
| JP Afumi pyrG For | TGCCTCAAACAATGCTCTTC | *A. fumigatus pyrG* |
| JP Afumi pyrG Rev | CCAGGTATCGTCGGGAGGT | *A. fumigatus pyrG* |
| JP AN2165 5' Flank For | CGGAGTCTGGGTTGAATCGC | 5’ Flank AN2165 |
| JP AN2165 5' Flank Rev | CGAAGAGGGTGAAGAGCATTGTTTGAGGCACAGCAGGAAGGAGGGCGAAG | 5’ Flank AN2165 |
| JP AN2165 3' Flank For | CACTGCTGTTATGGAGGGCTGCTATGGACCCTTTCCTACATCTGGGCGAC | 3’ Flank AN2165 |
| JP AN2165 3' Flank Rev | GTGACGACAATACCTCCCGACGATACCTGGCTTTCCTACATCTGGGCGAC | 3’ Flank AN2165 |
| JP AN2165 Nest For | GCGTGACTTGTGGACCGAAT | AN2165 KO Product |
| JP AN2165 Nest Rev | GGCGAAGAAGCAAAGAAGGA | AN2165 KO Product |
| JP AN5416 5' Flank For | TGCTGTGGACTATGGGGGA | 5’ Flank AN5416 |
| JP AN5416 5' Flank Rev | CGAAGAGGGTGAAGAGCATTGTTTGAGGCAGTAGGGGGAGGTATGGGAGA | 5’ Flank AN5416 |
| JP AN5416 3' Flank For | GTGACGACAATACCTCCCGACGATACCTGGAGCCGTTGTAATGTGAAGCC | 3’ Flank AN5416 |
| JP AN5416 3' Flank Rev | TCCTGTTCCCAAATACCACG | 3’ Flank AN5416 |
| JP AN5416 Nest For | GAGGGCGATGGATGTAGAGG | AN5416 KO Product |
| JP AN5416 Nest Rev | GCGGCTGAGACCTCTAATG | AN5416 KO Product |
| JP AN5874 5' Flank For | CCAGAAGAACAAATGACCGC | 5’ Flank AN5874 |
| JP AN5874 5' Flank Rev | CGAAGAGGGTGAAGAGCATTGTTTGAGGCACTTCTGACTGCCTTACGCC | 5’ Flank AN5874 |
| JP AN5874 3' Flank For | GTGACGACAATACCTCCCGACGATACCTGGTCACCAAACTGCTCACTTCC | 3’ Flank AN5874 |
| JP AN5874 3' Flank Rev | ACCCCCTCGCTATTTCAATC | 3’ Flank AN5874 |
| JP AN5874 Nest For | TCGCTATAAATGGTCCCTCG | AN5874 KO Product |
| JP AN5874 Nest Rev | TCTGTTTGTGTTGGCTTCCG | AN5874 KO Product |
| JP AN6749 5' Flank For | CACAATCAGGACGGTCACA | 5’ Flank AN6749 |
| JP AN6749 5' Flank Rev | CGAAGAGGGTGAAGAGCATTGTTTGAGGCATAGGTGGTAACGGGTTGAGG | 5’ Flank AN6749 |
| JP AN6749 3' Flank For | GTGACGACAATACCTCCCGACGATACCTGGCATTCGCCGCACTATACCCT | 3’ Flank AN6749 |
| JP AN6749 3' Flank Rev | ACATTCACTGGATAGGCGGG | 3’ Flank AN6749 |
| JP AN6749 Nest For | GGAGGTCAGAGAGCGAGGAG | AN6749 KO Product |
| JP AN6749 Nest Rev | GATAGGCGGGGTGAGAGTGG | AN6749 KO Product |
| JP AN7933 5' Flank For | GTTTCCCATAAGTCGGCATC | 5’ Flank AN7933 |
| JP AN7933 5' Flank Rev | CGAAGAGGGTGAAGAGCATTGTTTGAGGCATGCTGCCATTGCTTGTCCTC | 5’ Flank AN7933 |
| JP AN7933 3' Flank For | GTGACGACAATACCTCCCGACGATACCTGGCTGTGAAAACCCCTACCGA | 3’ Flank AN7933 |
| JP AN7933 3' Flank Rev | GGAGGTGTCGGACTGCGTG | 3’ Flank AN7933 |
| JP AN7933 Nest For | CGGTAGATGCCCAGTCCCAG | AN7933 KO Product |
| JP AN7933 Nest Rev | CGCAAAGCACCTAATCAACC | AN7933 KO Product |
| JP AN8833 5' Flank For | CTGAGGTGATGCTTCGGTG | 5’ Flank AN8833 |
| JP AN8833 5' Flank Rev | CGAAGAGGGTGAAGAGCATTGTTTGAGGCAACCCTGACGCAAAGAAAGAC | 5’ Flank AN8833 |
| JP AN8833 3' Flank For | GTGACGACAATACCTCCCGACGATACCTGGCTAAAGCCGAAAACGAGCAA | 3’ Flank AN8833 |
| JP AN8833 3' Flank Rev | CCCAATCAACAAAAAGCACG | 3’ Flank AN8833 |
| JP AN8833 Nest For | CAGCGAGCGAAGTCAGAAG | AN8833 KO Product |
| JP AN8833 Nest Rev | CAACTCAACCCTTCACCACG | AN8833 KO Product |
| JP AN8945 5' Flank For | CGGCTGGAAAAAAAGTACG | 5’ Flank AN8945 |
| JP AN8945 5' Flank Rev | CGAAGAGGGTGAAGAGCATTGTTTGAGGCAAAAATGTGGAGGGAGCGATA | 5’ Flank AN8945 |
| JP AN8945 3' Flank For | GTGACGACAATACCTCCCGACGATACCTGGGGCTTATTGTGGCTTCAGTC | 3’ Flank AN8945 |
| JP AN8945 3' Flank Rev | ATAAATCACATCTGCTCTCGTC | 3’ Flank AN8945 |
| JP AN8945 Nest For | TATGCGGGGTGATTGAGATT | AN8945 KO Product |
| JP AN8945 Nest Rev | GGGGCACAGGATTGAGGTTC | AN8945 KO Product |
| JP AN9193 5' Flank For | GGAGTCGCAAGCAGAAGAAT | 5’ Flank AN9193 |
| JP AN9193 5' Flank Rev | CGAAGAGGGTGAAGAGCATTGTTTGAGGCATATTAGGAGGTTTGAAGGC | 5’ Flank AN9193 |
| JP AN9193 3' Flank For | GTGACGACAATACCTCCCGACGATACCTGGGACTCGCCGTACCAACCACT | 3’ Flank AN9193 |
| JP AN9193 3' Flank Rev | TCCAGTCCACGACATTCCTA | 3’ Flank AN9193 |
| JP AN9193 Nest For | GGAGGAGAAGGAGAGAGAG | AN9193 KO Product |
| JP AN9193 Nest Rev | TTATGAGGGTTGATTCCGTG | AN9193 KO Product |
| JP veA S-tag 5’ Flank For | CCTTTATCGTCTACAGCGCC | 5’ Flank veA-Stag |
| JP veA S-tag 5’ Flank Rev | TTCTTTGGCTCCAGCGCCTGCACCAGCTCCACGCATGGTGGCAGGCTTTG | 5’ Flank veA-Stag |
| JP veA S-tag 3’ Flank For | CATCACGCATCAGTGCCTCCTCTCAGACAGTAAAAGAATTCTGCCGGCGT | 3’ Flank veA-Stag |
| JP veA S-tag 3’ Flank Rev | CGATTCGTTTCGAAGTTGCGC | 3’ Flank veA-Stag |
| JP gpdA(p) For (EcoRI) | TTTCGAATTCCATCCGGATGTCGAAGG | *A. nidulans gpdA* promoter |
| JP OE llmF Fusion Rev | GTCGTCGCTGTATTGGGTCATGGTGATGTCTGCTCAAG | *A. nidulans gpdA* promoter |
| JP OE llmF Fusion For | CTTGAGCAGACATCACCATGACCCAATACAGCGACGAC | *llmF* gDNA ORF |
| JP OE llmF Rev (NotI) | TGCAAGGCGGCCGCTGCCAATGGAGTGCATA | *llmF* gDNA ORF |
| JP LlmF-GFP C-term For | GGGTTCGGGGATATCAAGTCCGCCTCGCGGAGCTGGTGCAGGCGCTG | *llmF*-GFP C-tag |
| JP LlmF-GFP C-term Rev | GCTCGCGCCAAGATGCTAGATTGGGAATTATTTGTATAGTTCATCC | *llmF*-GFP C-tag |
| JP GFP-LlmF N term For | CAGCTACCCCGCTTGAGCAGACATCACCATGTCCAAGGGCGAGGAACT | GFP-*llmF* N tag |
| JP GFP-LlmF N term Rev | ATCGATATCGTCGTCGCTGTATTGGGTCATGGCTCCAGCGCCTGCACC | GFP-*llmF* N tag |
| JP TAP-LlmF N term For | CAGCTACCCCGCTTGAGCAGACATCACCATGGCAGGCCTTGCGCAAC | TAP-LlmF tag |
| JP TAP-LlmF N term Rev | CATGGCTCCAGCGCCTGCACCAGCTCCGTCGACGGTATCGATAAGCTT | TAP-LlmF tag |
| JP LlmF SAM mut For | GGAAATCCTCGACGTCGCTACCGCGACCGGAATCTGGGCAATG | LlmF^SAM^ |
| JP LlmF SAM mut Rev | CATTGCCCAGATTCCGGTCGCGGTAGCGACGTCGAGGATTTCC | LlmF^SAM^ |
| JP AN2165 Internal For | CAGGTTACCTCTTCACGG | *llmA* northern probe |
| JP RT-PCR AN2165 Rev | CTATCCATCCACCCAGCAA | *llmA* northern probe |
| JP RT-PCR AN8945 For | AGCCTTCAAATCGCTCAAAC | *llmB* northern probe |
| JP AN8945 Internal Rev | CTACTCCGGCTTCTGCCCAT | *llmB* northern probe |
| JP RT-PCR AN7933 For | TACGAGAAGCAGTGGACGTG | *llmC* northern probe |
| JP AN7933 Internal Rev | CACGTCTCGGTAGGGGTTTTC | *llmC* northern probe |
| JP AN5416 Internal Rev | CACAAGTCATGCATCATATG | *llmD* northern probe |
| JP RT-PCR AN5416 Rev | CTTCAAGGAGCCATCCTCAG | *llmD* northern probe |
| JP AN6749 GFP 5' For | ATCCTCGACGTCGGTACC | *llmF* northern probe |
| JP RT-PCR AN6749 Rev | AACCGCTGTTCCACTACGTC | *llmF* northern probe |
| JP AN5874 Internal For | GTAGATGATCTAAACCGCC | *llmG* northern probe |
| JP RT-PCR AN5874 Rev | CACGCGGATAAAGGTAGAGG | *llmG* northern probe |
| JP AN8833 Internal For | GATATTGTAACGACACATACTTC | *llmI* northern probe |
| JP RT-PCR AN8833 Rev | GTTTGCGTGCTGTCACTGTT | *llmI* northern probe |
| JP AN9193 Internal For | AGGATATGGCTATGGGATGCC | *llmJ* northern probe |
| JP RT-PCR AN9193 Rev | TGTTTAAGAGCCCAGCATCC | *llmJ* northern probe |
| 07253-5'AN6749NcoI | AACCATGGAAATGACCCAATACAGCGACGAC | Clone AN6749 (llmF) |
| 07254-3'AN6749BamHI | AAGGATCCTCAGCGAGGCGGACTTGATATCCCC | Clone AN6749 (llmF) |
| JP VeA For (NotI) | TATGCAGCGGCCGCAATGGCTACACTTGCAGCAC | Y2H veA truncation |
| JP VeA Rev (PstI) | CTATGACTGCAGTTAACGCATGGTGGCAGGCT | Y2H veA truncation |
| JP VeA (29) For (NotI) | TATGCAGCGGCCGCAAAGATTACCTATAAATTGAAT | Y2H veA truncation |
| JP VeA (235) Rev (PstI) | CTATGACTGCAGTTACCGCACATCGCGCCGGATAC | Y2H veA truncation |
| JP VeA (236) For (NotI) | TATGCAGCGGCCGCAATGAGACGGCGCGGAGACAA | Y2H veA truncation |
| JP veA (458) For (NotI) | TATGCAGCGGCCGCATCGAAAACACCATCTAATATG | Y2H veA truncation |
| JP LlmF (90) Rev (PstI) | TAGCTACTGCAGTCAGTCGAGGATTTCCTGCGGG | Y2H llmF truncation |
| JP LlmF (180) Rev (PstI) | TAGCTACTGCAGTCATACAACCTCGATGTATCCG | Y2H llmF truncation |
| JP LlmF (80) For (EcoRI) | ATATCGGAATTCCCGATCGAGAACCCGCAGG | Y2H llmF truncation |
| JP LlmF (180) For (EcoRI) | ATATCGGAATTCGTAGAACACTCCGTTCAGC | Y2H llmF truncation |
